# Supplementary material for: Transcriptome Analysis of Clinopodium chinense (Benth.) O. Kuntze and Identification of Genes Involved in Triterpenoid Saponin Biosynthesis
Source: Int J Mol Sci. 2019 May 29;20(11):2643. doi: 10.3390/ijms20112643 (PMC6600151; doi:10.3390/ijms20112643)
Supplement: Supplementary file 1 [file ijms-20-02643-s001.pdf]

Transcriptome analysis of *Clinopodium chinense* (Benth.) O. Kuntze and identification of genes involved in triterpenoid saponin biosynthesis

Yuanyuan Shi[1,2], Shengxiang Zhang[1,2], Daiyin Peng[1,3], Chenkai Wang[1,2], Derui Zhao[1,2], Kelong Ma[1,4], Jiawen Wu[1,2,3*], Luqi Huang[1,5*]

[1] Anhui University of Chinese Medicine and Anhui Academy of Chinese Medicine, Hefei 230038,China.

871735748@qq.com (Y.S.); 351038096@qq.com (S.Z.); pengdy@ahtcm.edu.cn (D.P.); 846981810@qq.com (C.W.); 920031823@qq.com (D.Z.); 515587551@qq.com (K.M.); wujiawen@ahtcm.edu.cn (J.W.); huangluqi01@126.com (L.H.).

[2] Key Laboratory of Xin'an Medicine, Ministry of Education, Anhui University of Chinese Medicine, Hefei 230038, China.

[3] Synergetic Innovation Center of Anhui Authentic Chinese Medicine Quality Improvement, Hefei 230012, China.

[4] Clinical College of Integrated Traditional Chinese and Western Medicine, Anhui University of Chinese Medicine.

[5] State Key Laboratory Breeding Base of Dao-di Herbs，National Resource Center for Chinese Materia Medica, China Academy of Chinese Medical Sciences, Beijing 100700, China.

* Corresponding author. Jiawen Wu, Luqi Huang, E-mail address: wujiawen@ahtcm.edu.cn, huangluqi01@126.com.

Supplementary data

Figure S1 Total saponin contents from leaves, stems, and roots of *C. chinense*.

Figure S2 The length distribution of unigenes for *C. chinense* transcriptome assembly.

Figure S3 Annotation and Blast results for the *C. chinense* transcripts and unigenes.

Figure S4 Annotation of unigenes in *C. chinense* using MAPMAN software.

Figure S5 Overview of metabolic pathway mapped with selected unigenes (FPKM > 1) using MAPMAN analysis.

Figure S6 Overview of secondary metabolic pathway mapped with selected unigenes (FPKM > 1) using MAPMAN analysis.

Figure S7 GO function annotation of *C. chinense* unigene.

Figure S8 KEGG function annotation of *C. chinense* unigene.

Figure S9 Terpenoid backbone biosynthesis based on the KEGG database. Red box represents enzymes annotated by KEGG database.

Figure S10 Sesquiterpenoid and triterpenoid biosynthesis based on the KEGG database. Red

box represents enzymes annotated by KEGG database.

Figure S11 Steroid biosynthesis based on the KEGG database. Red box represents enzymes annotated by KEGG database.

Figure S12 Model summary of pathways involved in triterpenoid saponin biosynthesis.

Figure S13 Pathway for hormone biosynthesis in *C. chinense* using MAPMAN analysis.

Figure S14 Standard curve of Clinopodiside A at 250 nm.

Table S1 KEGG annotation of all unigenes.

Table S2 Cytochrome P450s annotated in *C. chinense*.

Table S3 Glucosyltransferases annotated in *C. chinense*.

Table S4 Beta amyrin synthase annotated in *C. chinense*.

Table S5 GO terms from biological process ontology for leaf versus root in *C. chinense*.

Table S6 GO terms from biological process ontology for stem versus root in *C. chinense*.

Table S7 GO terms from molecular function ontology for leaf versus root in *C. chinense*.

Table S8 GO terms from molecular function ontology for stem versus root in *C. chinense*.

Table S9 RNA information of different tissues.

Table S10 Genes and primer sequences used for qRT-PCR.

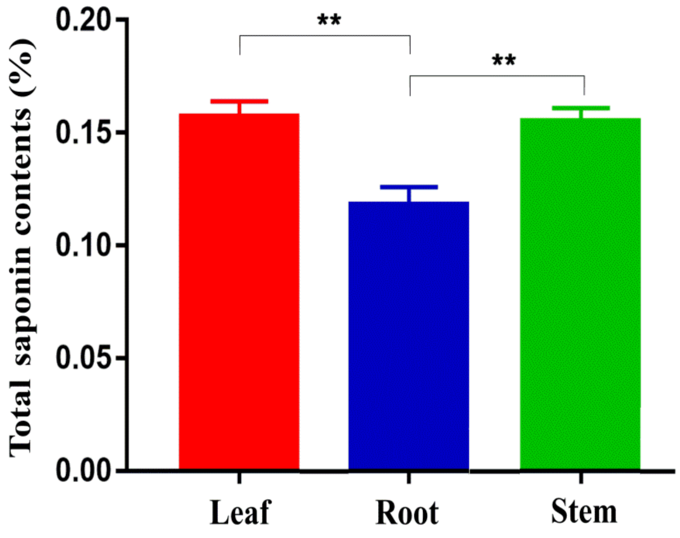

Supplementary Figure S1 Total saponin contents from leaves, stems, and roots of *C. chinense*. Total saponin contents of root, leaf, and stem are colored in blue, red, and green, respectively. ** represents p-values less than 0.01, which was defined as a significant difference.

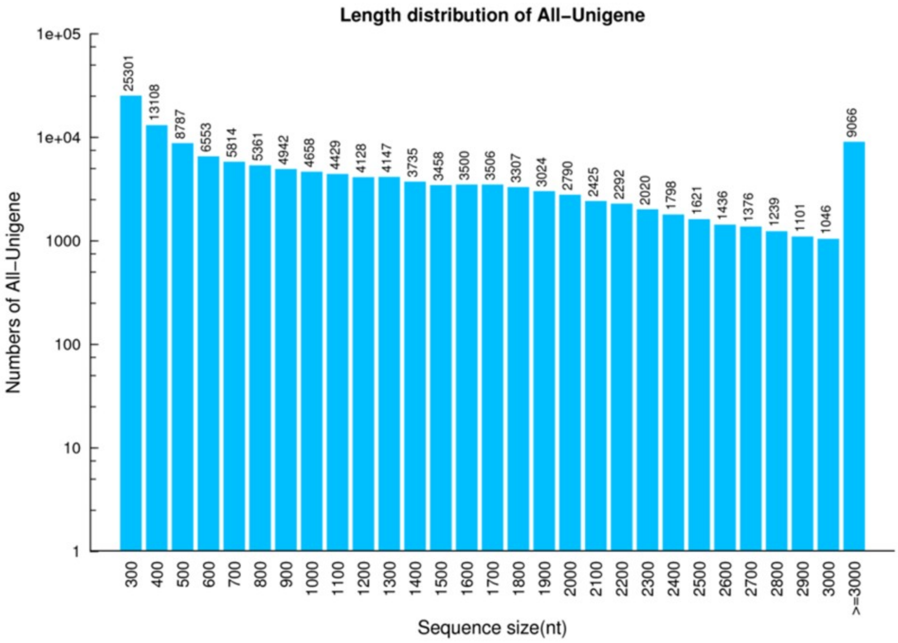

Supplementary Figure S2 The length distribution of unigenes for *C. chinense* transcriptome assembly.

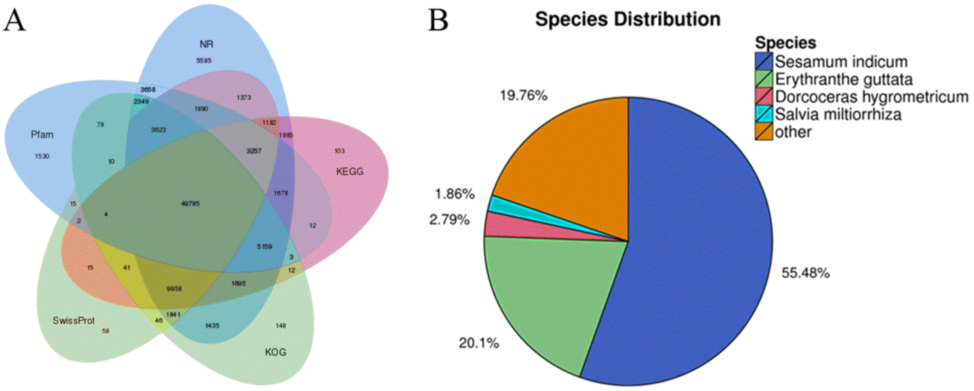

Supplementary Figure S3 Annotation and Blast results for the *C. chinense* transcripts and unigenes. (A) Transcripts and unigenes of *C. chinense* were annotated to the NR, KEGG, KOG, Swiss-Prot and Pfam databases. (B) The species distribution of *C. chinense* in NR database.

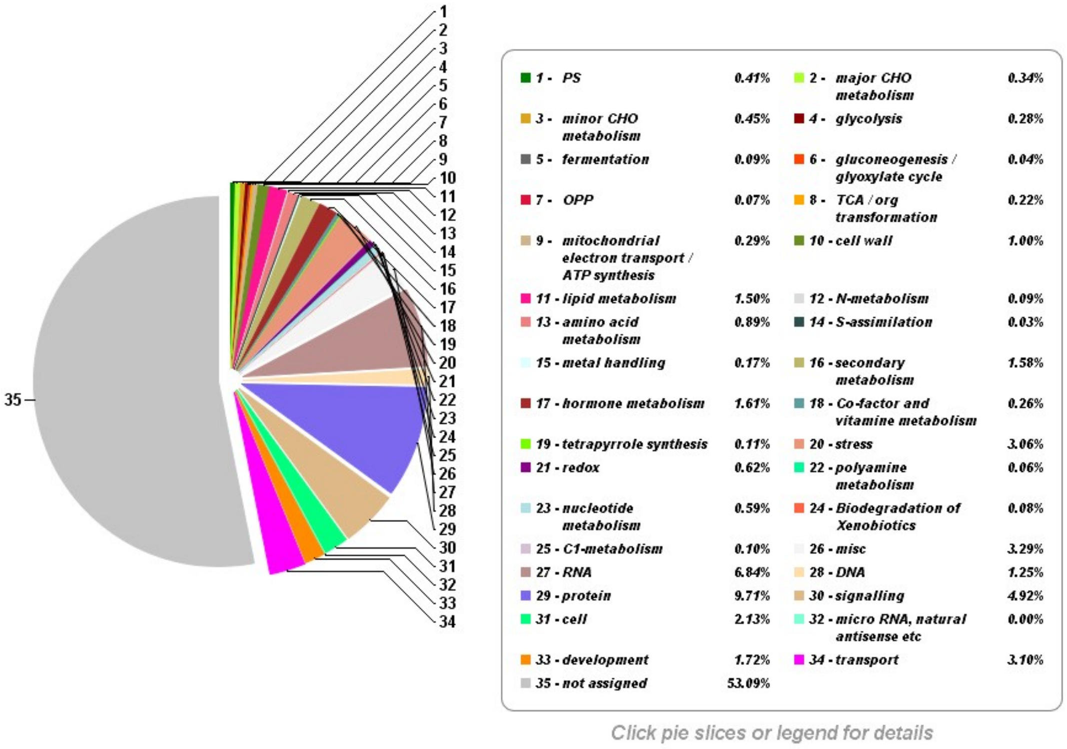

Supplementary Figure S4 Annotation of unigenes in *C. chinense* using MAPMAN software. Different metabolic pathways are shown in different colors. The annotated proportion of pathways are shown in the fan-shaped area.

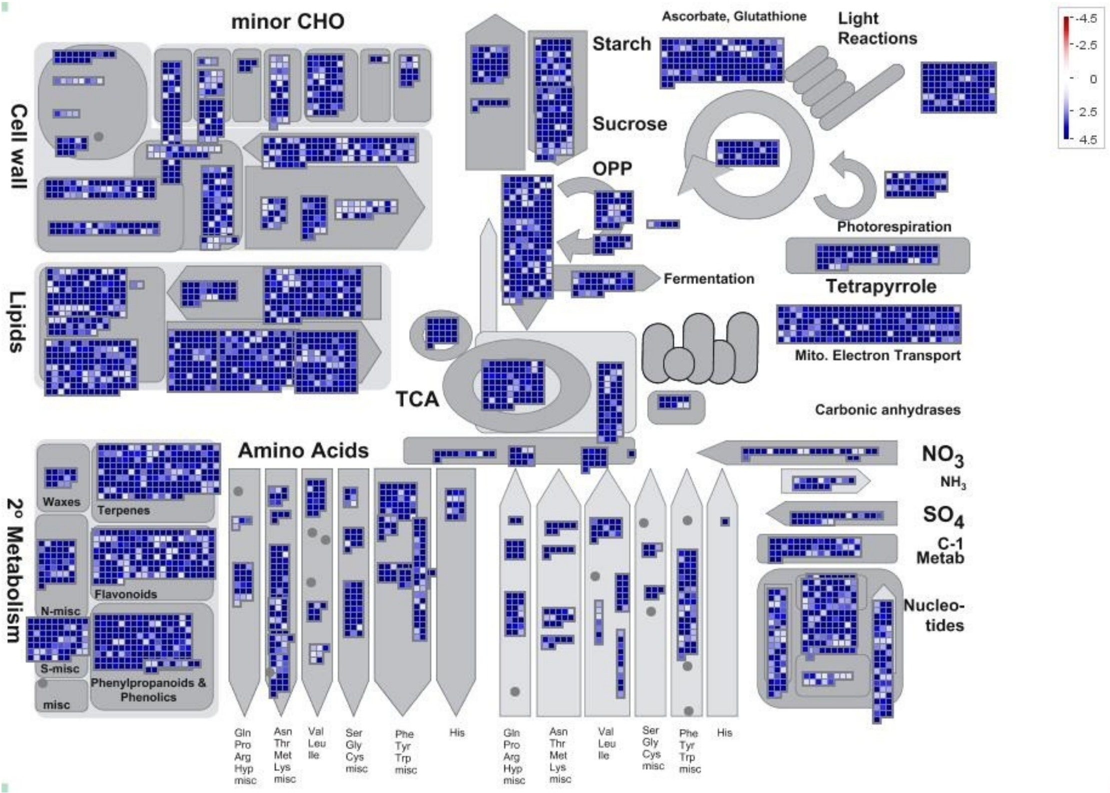

Supplementary Figure S5: Overview of metabolic pathway mapped with selected unigenes (FPKM > 1) using MAPMAN analysis. Blue and red represent high and low expression levels, respectively.

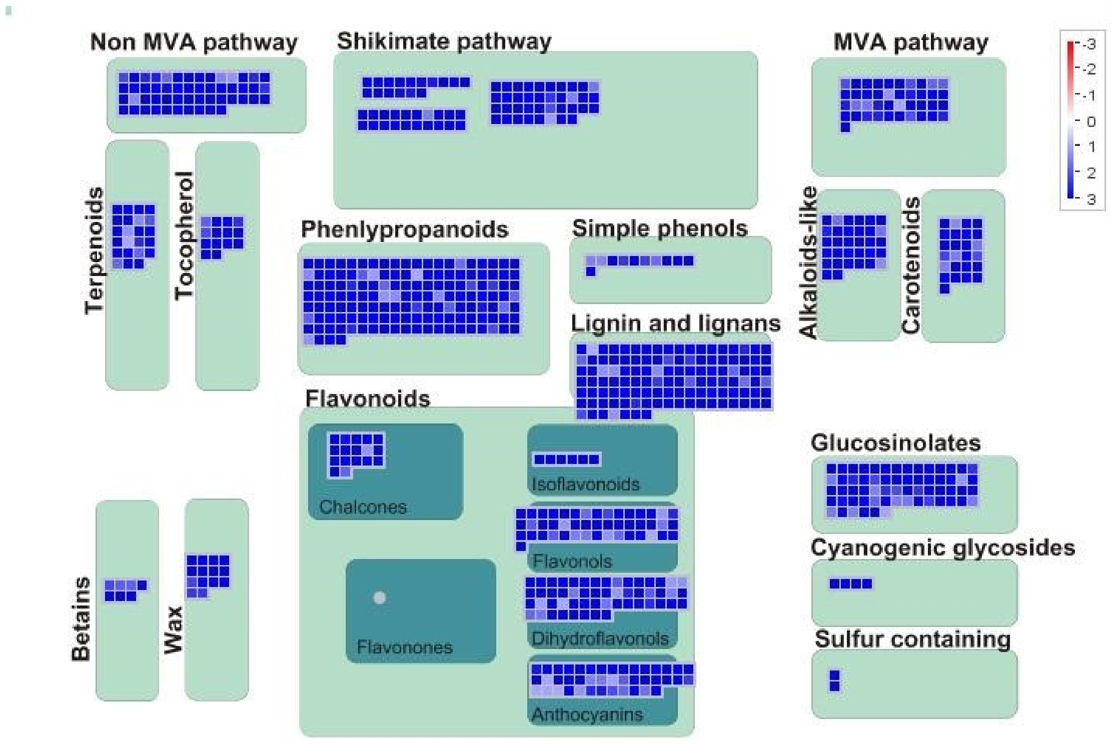

Supplementary Figure S6: Overview of secondary metabolic pathway mapped with selected unigenes (FPKM > 1) using MAPMAN analysis. Blue and red represent high and low expression levels, respectively.

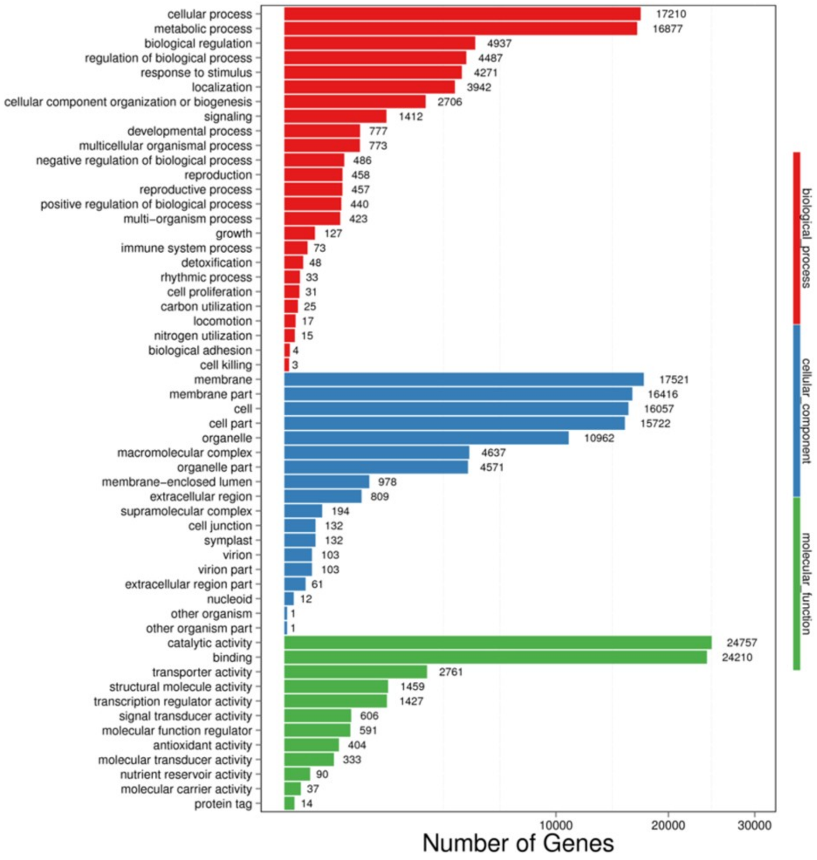

Supplementary Figure S7 GO function annotation of *C. chinense* unigenes. The annotated unigenes were described using GO terms and divided into three classes, biological process, cellular component, and molecular function, colored in red, blue, and green, respectively.

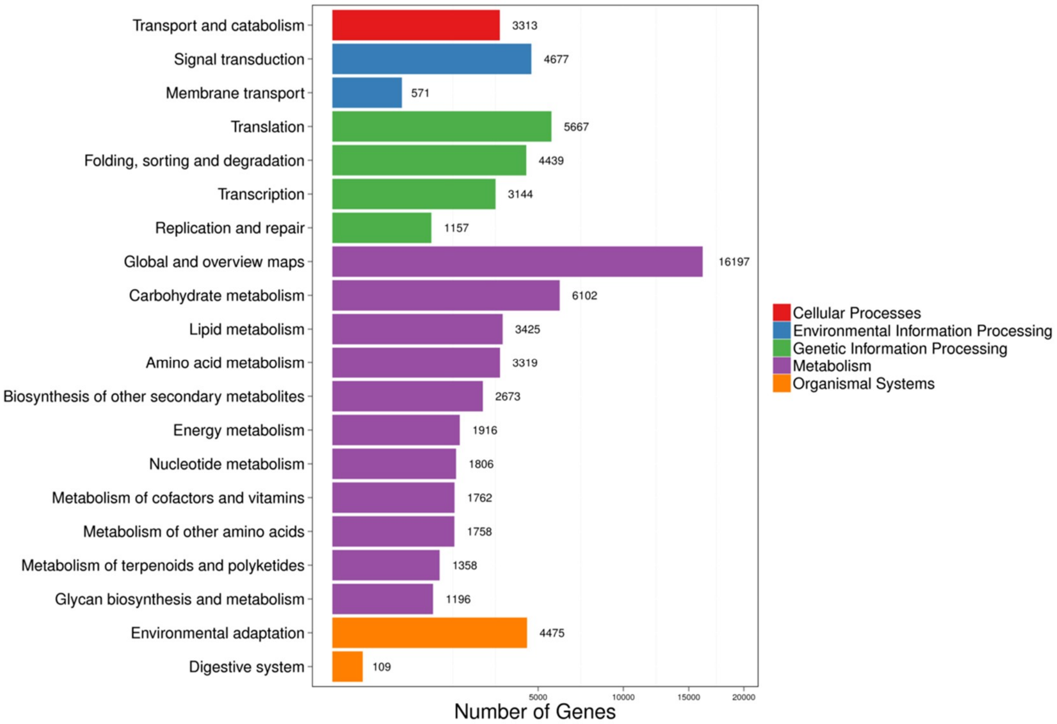

Supplementary Figure S8 KEGG function annotation of *C. chinense* unigenes. The annotated unigenes were mapped to the KEGG database and classified into five categories, cellular process, genetic information processing, metabolism, organismal systems, and environmental information processing, colored in red, green, purple, orange, and blue, respectively.

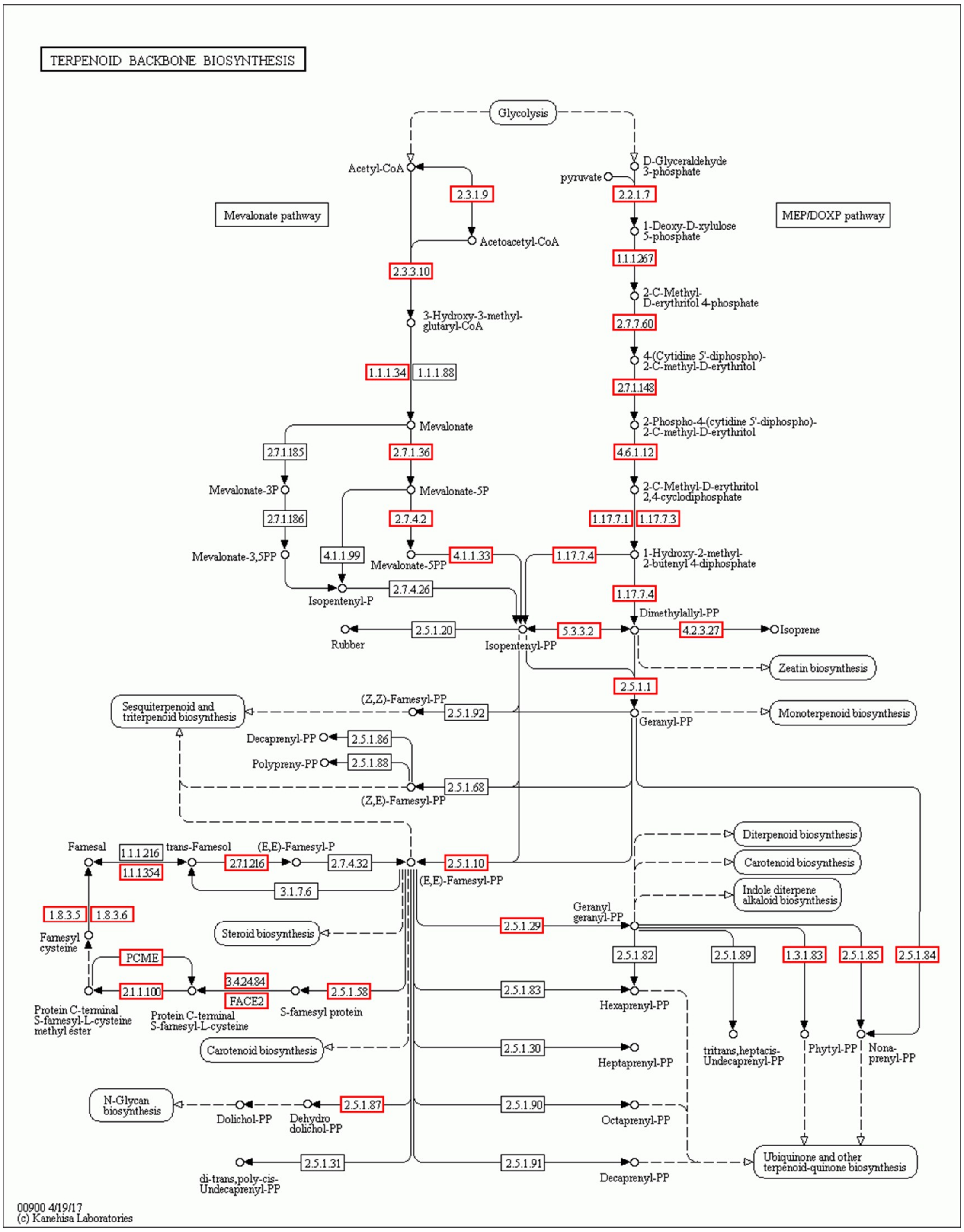

Supplementary Figure S9 Terpenoid backbone biosynthesis based on the KEGG database. Red box represents enzymes annotated by KEGG database.

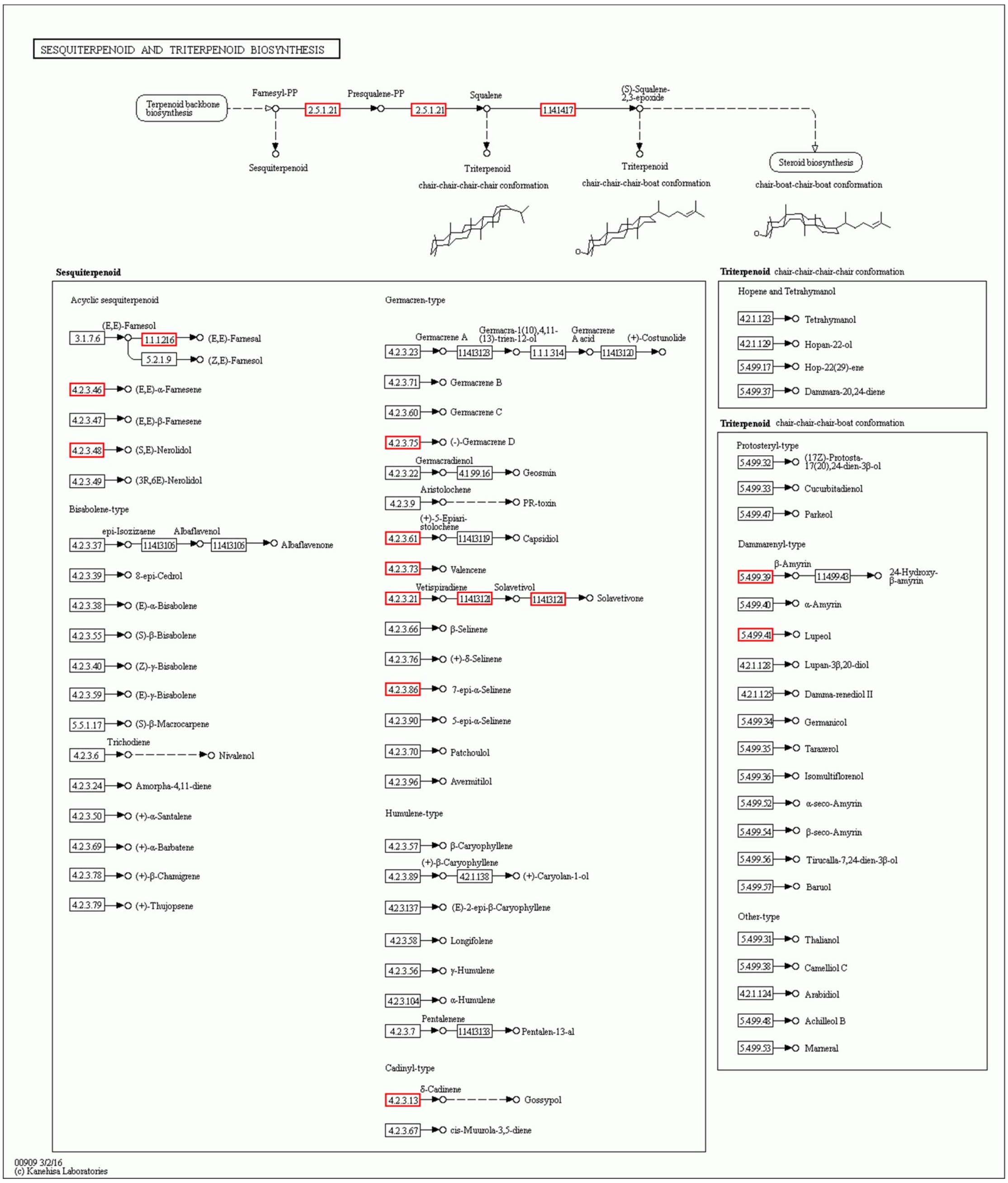

Supplementary Figure S10 Sesquiterpenoid and triterpenoid biosynthesis based on the KEGG database. Red box represents enzymes annotated by KEGG database.

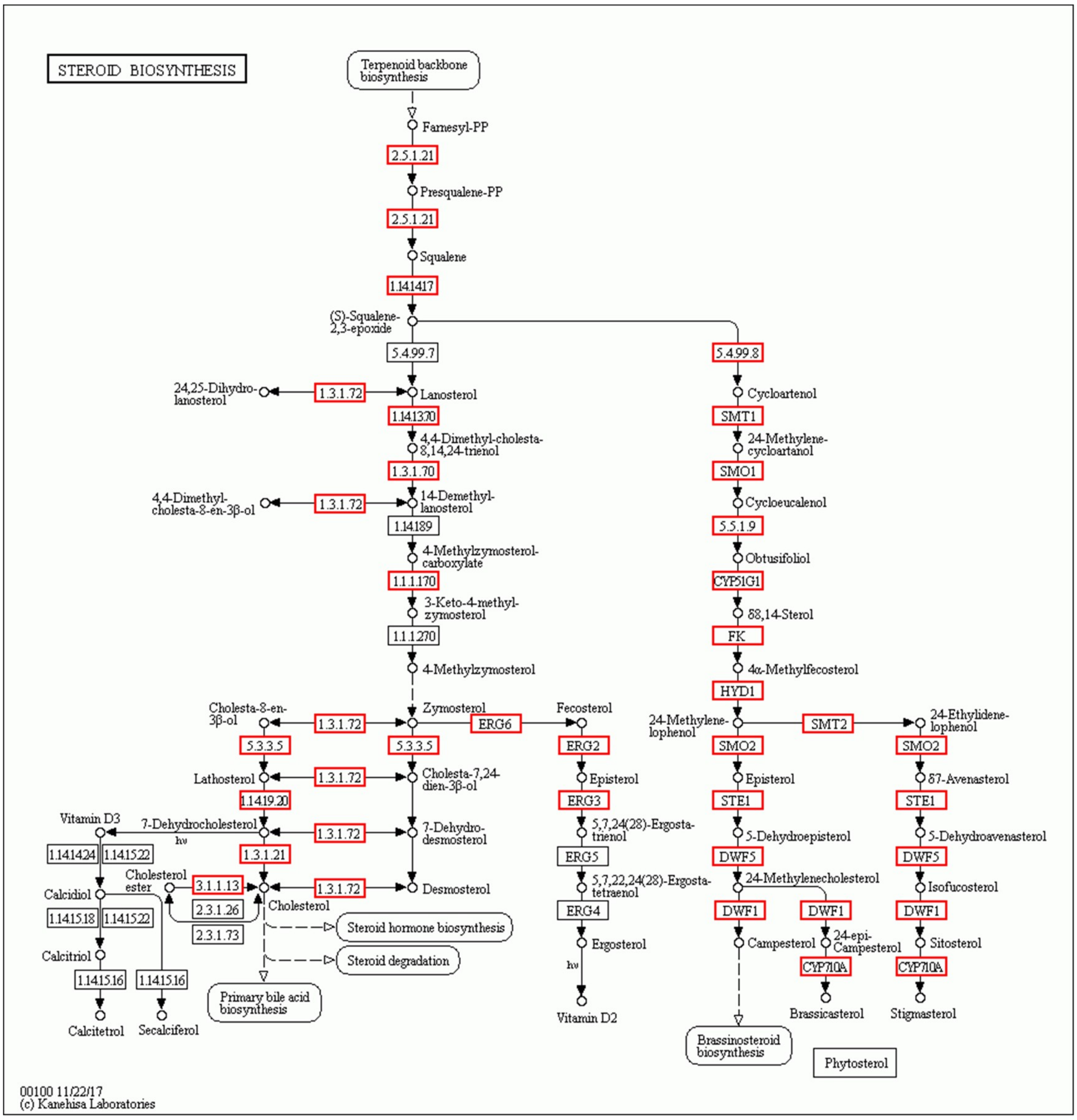

Supplementary Figure S11 Steroid biosynthesis based on the KEGG database. Red box represents enzymes annotated by KEGG database.

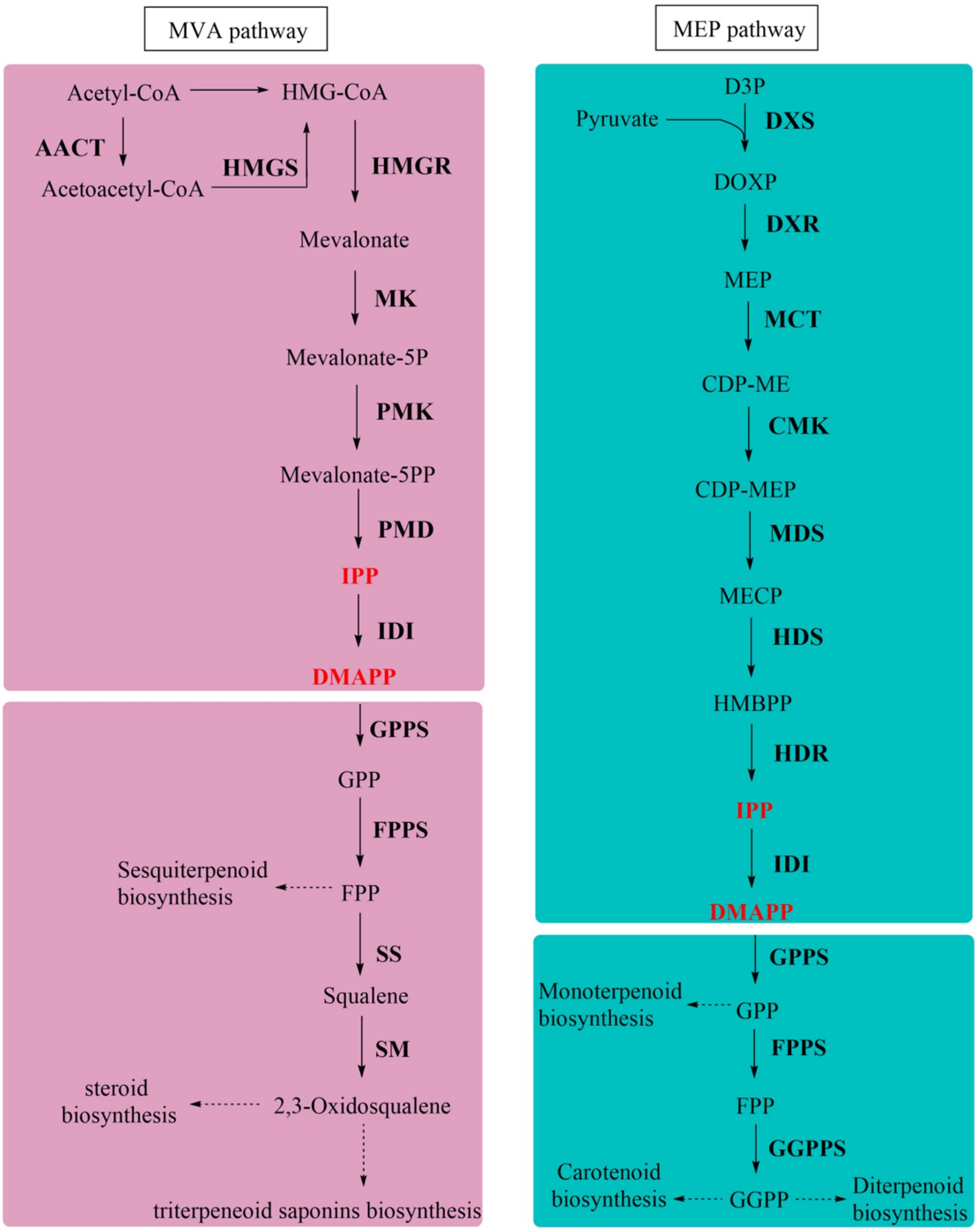

Supplementary Figure S12 Model summary of pathways involved in triterpenoid saponin biosynthesis. Triterpenoid saponins are synthesized by the MVA pathway in cytoplasm and mitochondria (shown in purple) or the MEP pathway in plastids (shown in blue). IPP and DMAPP (shown in red) are the precursors of isoprenoids, including monoterpenoids, sesquiterpenoids, diterpenoids, triterpenoid saponins, steroids and caroteroids.

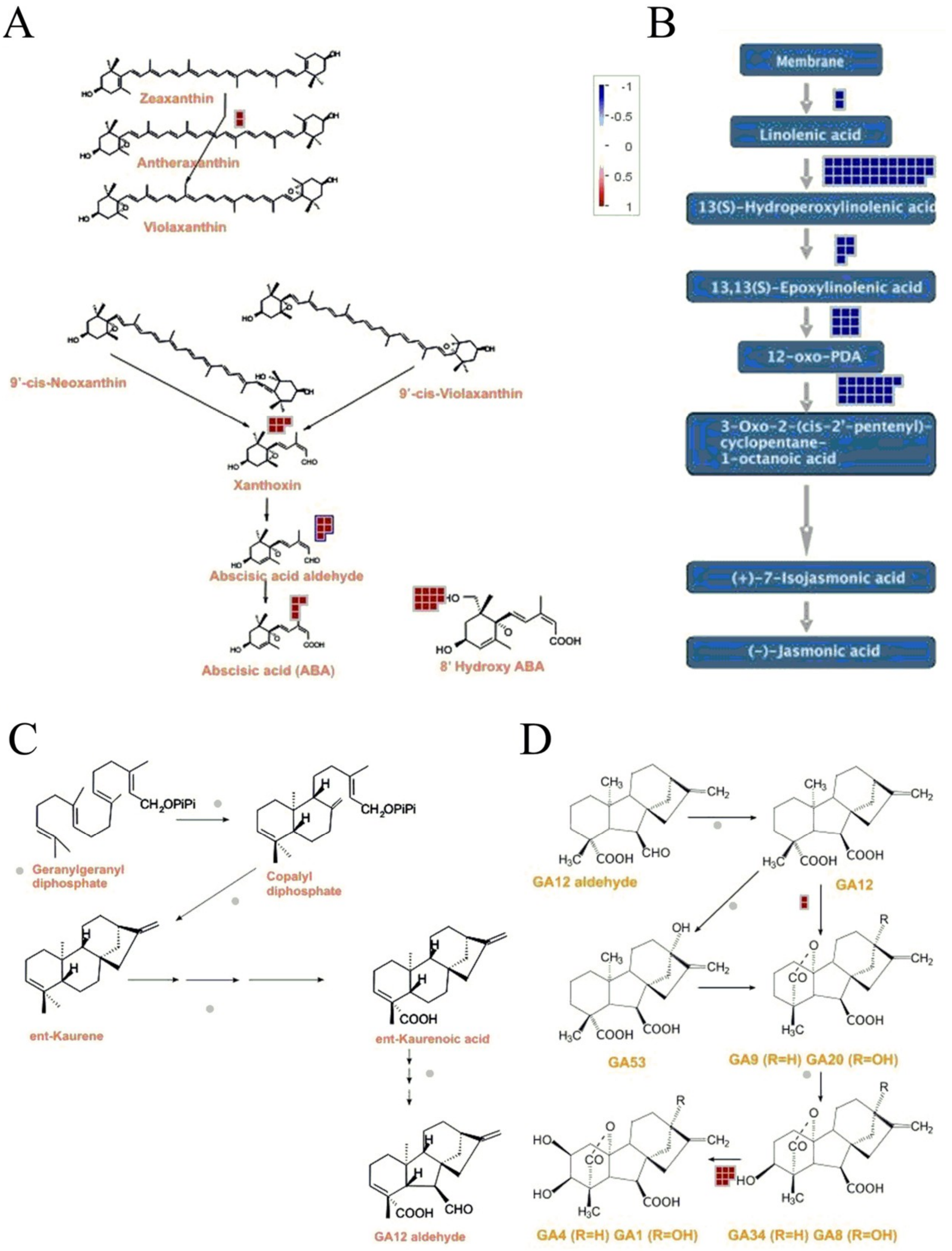

Supplementary Figure S13 Pathway for hormone biosynthesis in *C. chinense* using MAPMAN analysis. (A) Pathway for abscisic acid (ABA) biosynthesis. (B) Pathway for jasmonate (JA) biosynthesis. (C) and (D) Pathway for gibberellin (GA) biosynthesis.

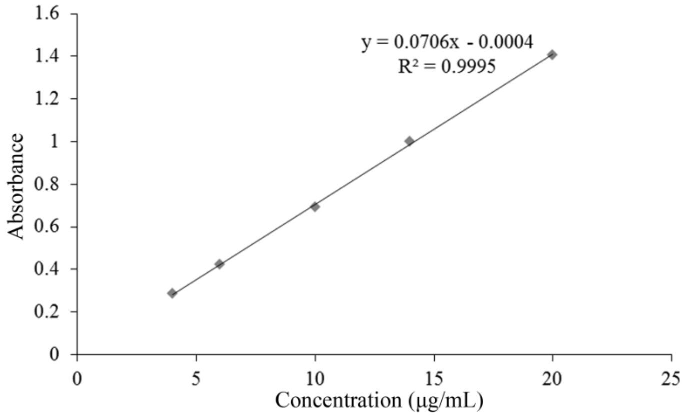

Supplementary Figure S14 Standard curve of Clinopodiside A at 250 nm.

Supplementary Table S1 KEGG annotation of unigenes.

Pathway	Unigene number	Pathway ID
Metabolic pathways	15096 (20.18%)	ko01100
Biosynthesis of secondary metabolites	8307 (11.11%)	ko01110
Plant-pathogen interaction	3925 (5.25%)	ko04626
MAPK signaling pathway - plant	2522 (3.37%)	ko04016
Plant hormone signal transduction	2407 (3.22%)	ko04075
RNA transport	2090 (2.79%)	ko03013
Spliceosome	2026 (2.71%)	ko03040
Ribosome	1831 (2.45%)	ko03010
Endocytosis	1787 (2.39%)	ko04144
Protein processing in endoplasmic reticulum	1746 (2.33%)	ko04141
Biosynthesis of amino acids	1669 (2.23%)	ko01230
Phenylpropanoid biosynthesis	1655 (2.21%)	ko00940
Starch and sucrose metabolism	1622 (2.17%)	ko00500
Carbon metabolism	1593 (2.13%)	ko01200
Purine metabolism	1503 (2.01%)	ko00230
mRNA surveillance pathway	1391 (1.86%)	ko03015
Pyrimidine metabolism	1324 (1.77%)	ko00240
Ubiquitin mediated proteolysis	1169 (1.56%)	ko04120
RNA degradation	993 (1.33%)	ko03018
Amino sugar and nucleotide sugar metabolism	962 (1.29%)	ko00520
Glycolysis / Gluconeogenesis	872 (1.17%)	ko00010
Glycerophospholipid metabolism	818 (1.09%)	ko00564
Galactose metabolism	811 (1.08%)	ko00052
Peroxisome	780 (1.04%)	ko04146
Glycerolipid metabolism	779 (1.04%)	ko00561
RNA polymerase	774 (1.03%)	ko03020
Phosphatidylinositol signaling system	704 (0.94%)	ko04070
Oxidative phosphorylation	703 (0.94%)	ko00190
Cysteine and methionine metabolism	657 (0.88%)	ko00270
Ribosome biogenesis in eukaryotes	638 (0.85%)	ko03008
Pentose and glucuronate interconversions	629 (0.84%)	ko00040
Pyruvate metabolism	602 (0.8%)	ko00620
Ascorbate and aldarate metabolism	602 (0.8%)	ko00053
Phagosome	595 (0.8%)	ko04145
Glutathione metabolism	582 (0.78%)	ko00480

Inositol phosphate metabolism	581 (0.78%)	ko00562
ABC transporters	571 (0.76%)	ko02010
Cyanoamino acid metabolism	560 (0.75%)	ko00460
Circadian rhythm - plant	550 (0.74%)	ko04712
Fatty acid metabolism	529 (0.71%)	ko01212
Aminoacyl-tRNA biosynthesis	506 (0.68%)	ko00970
Arginine and proline metabolism	477 (0.64%)	ko00330
Flavonoid biosynthesis	468 (0.63%)	ko00941
Sphingolipid metabolism	465 (0.62%)	ko00600
Nucleotide excision repair	449 (0.6%)	ko03420
Tryptophan metabolism	448 (0.6%)	ko00380
Glyoxylate and dicarboxylate metabolism	446 (0.6%)	ko00630
Valine, leucine and isoleucine degradation	431 (0.58%)	ko00280
Fatty acid degradation	428 (0.57%)	ko00071
2-Oxocarboxylic acid metabolism	423 (0.57%)	ko01210
Pentose phosphate pathway	417 (0.56%)	ko00030
Carbon fixation in photosynthetic organisms	417 (0.56%)	ko00710
Glycine, serine and threonine metabolism	412 (0.55%)	ko00260
N-Glycan biosynthesis	409 (0.55%)	ko00510
Fructose and mannose metabolism	409 (0.55%)	ko00051
Homologous recombination	399 (0.53%)	ko03440
Phenylalanine, tyrosine and tryptophan biosynthesis	386 (0.52%)	ko00400
Alanine, aspartate and glutamate metabolism	384 (0.51%)	ko00250
alpha-Linolenic acid metabolism	382 (0.51%)	ko00592
Basal transcription factors	363 (0.49%)	ko03022
DNA replication	361 (0.48%)	ko03030
Phenylalanine metabolism	361 (0.48%)	ko00360
Tyrosine metabolism	358 (0.48%)	ko00350
Lysine degradation	356 (0.48%)	ko00310
beta-Alanine metabolism	352 (0.47%)	ko00410
Terpenoid backbone biosynthesis	345 (0.46%)	ko00900
Proteasome	341 (0.46%)	ko03050
Other glycan degradation	337 (0.45%)	ko00511
Ubiquinone and other terpenoid-quinone biosynthesis	335 (0.45%)	ko00130
Protein export	322 (0.43%)	ko03060
Carotenoid biosynthesis	315 (0.42%)	ko00906
Base excision repair	310 (0.41%)	ko03410
Citrate cycle (TCA cycle)	308 (0.41%)	ko00020

Arginine biosynthesis	307 (0.41%)	ko00220
Biosynthesis of unsaturated fatty acids	305 (0.41%)	ko01040
Arachidonic acid metabolism	302 (0.4%)	ko00590
Ether lipid metabolism	300 (0.4%)	ko00565
Nitrogen metabolism	297 (0.4%)	ko00910
Porphyrin and chlorophyll metabolism	283 (0.38%)	ko00860
Mismatch repair	272 (0.36%)	ko03430
Propanoate metabolism	267 (0.36%)	ko00640
Diterpenoid biosynthesis	267 (0.36%)	ko00904
Stilbenoid, diarylheptanoid and gingerol biosynthesis	252 (0.34%)	ko00945
Photosynthesis	247 (0.33%)	ko00195
Autophagy - other	240 (0.32%)	ko04136
Sulfur metabolism	240 (0.32%)	ko00920
Pantothenate and CoA biosynthesis	234 (0.31%)	ko00770
Isoquinoline alkaloid biosynthesis	230 (0.31%)	ko00950
Linoleic acid metabolism	229 (0.31%)	ko00591
Fatty acid biosynthesis	221 (0.3%)	ko00061
Glycosaminoglycan degradation	216 (0.29%)	ko00531
SNARE interactions in vesicular transport	213 (0.28%)	ko04130
Steroid biosynthesis	197 (0.26%)	ko00100
Indole alkaloid biosynthesis	197 (0.26%)	ko00901
Folate biosynthesis	194 (0.26%)	ko00790
Glycosylphosphatidylinositol (GPI)-anchor biosynthesis	192 (0.26%)	ko00563
Cutin, suberine and wax biosynthesis	190 (0.25%)	ko00073
Selenocompound metabolism	184 (0.25%)	ko00450
Fatty acid elongation	183 (0.24%)	ko00062
Tropane, piperidine and pyridine alkaloid biosynthesis	179 (0.24%)	ko00960
Nicotinate and nicotinamide metabolism	170 (0.23%)	ko00760
Butanoate metabolism	169 (0.23%)	ko00650
Sesquiterpenoid and triterpenoid biosynthesis	166 (0.22%)	ko00909
Glycosphingolipid biosynthesis - ganglio series	159 (0.21%)	ko00604
Histidine metabolism	155 (0.21%)	ko00340
Valine, leucine and isoleucine biosynthesis	149 (0.2%)	ko00290
Isoflavonoid biosynthesis	141 (0.19%)	ko00943
Riboflavin metabolism	141 (0.19%)	ko00740
Other types of O-glycan biosynthesis	135 (0.18%)	ko00514
One carbon pool by folate	135 (0.18%)	ko00670
Zeatin biosynthesis	115 (0.15%)	ko00908

Anthocyanin biosynthesis	114 (0.15%)	ko00942
Vitamin B6 metabolism	114 (0.15%)	ko00750
Monoterpenoid biosynthesis	111 (0.15%)	ko00902
Cholesterol metabolism	109 (0.15%)	ko04979
Thiamine metabolism	107 (0.14%)	ko00730
Biotin metabolism	107 (0.14%)	ko00780
Flavone and flavonol biosynthesis	105 (0.14%)	ko00944
Phosphonate and phosphinate metabolism	95 (0.13%)	ko00440
Glycosphingolipid biosynthesis - globo and isoglobo series	89 (0.12%)	ko00603
C5-Branched dibasic acid metabolism	80 (0.11%)	ko00660
Brassinosteroid biosynthesis	78 (0.1%)	ko00905
Lysine biosynthesis	78 (0.1%)	ko00300
Glucosinolate biosynthesis	78 (0.1%)	ko00966
Monobactam biosynthesis	71 (0.09%)	ko00261
Sulfur relay system	63 (0.08%)	ko04122
Taurine and hypotaurine metabolism	60 (0.08%)	ko00430
Photosynthesis - antenna proteins	59 (0.08%)	ko00196
Benzoxazinoid biosynthesis	58 (0.08%)	ko00402
Synthesis and degradation of ketone bodies	54 (0.07%)	ko00072
Non-homologous end-joining	49 (0.07%)	ko03450
Betalain biosynthesis	44 (0.06%)	ko00965
Mannose type O-glycan biosynthesis	21 (0.03%)	ko00515
Lipoic acid metabolism	17 (0.02%)	ko00785
Caffeine metabolism	11 (0.01%)	ko00232
Glycosphingolipid biosynthesis - lacto and neolacto series	11 (0.01%)	ko00601

Supplementary Table S2 Cytochrome P450s annotated in *C. chinense*.

Query_id	Subject_id	Identity	E_value	Subject_annotation
Unigene5892	XP_012852863.1	64.04	2.4E-194	cytochrome P450 [Theobroma cacao]
Unigene61313	AGN04218.1	64.31	1.1E-122	cytochrome P450 [Salvia miltiorrhiza]
CL10076.Contig2	KZV21240.1	67.69	3.2E-199	cytochrome P450 89A2 [Sesamum indicum]
CL11607.Contig1	XP_012850388.1	88.44	2.2E-171	cytochrome P450 [Ocimum basilicum]
CL12037.Contig2	XP_012074684.1	85.66	2.9E-266	cytochrome P450 98A2 [Jatropha curcas]
CL12037.Contig3	XP_012074684.1	86.77	3.8E-266	cytochrome P450 98A2 [Jatropha curcas]
CL13058.Contig1	AOW42546.1	69.7	3.8E-204	cytochrome P450 76AK8 [Salvia rosmarinus]
CL14718.Contig2	AOW42546.1	65.81	2.1E-192	cytochrome P450 76AK8 [Salvia rosmarinus]
CL3121.Contig1	XP_011072254.1	83.46	6.6E-259	cytochrome P450 734A1 [Sesamum indicum]
CL3121.Contig2	XP_012856870.1	70.08	6.8E-37	cytochrome P450 734A1 [Sesamum indicum]
CL3121.Contig3	XP_011072254.1	80.83	1.3E-179	cytochrome P450 734A1 [Sesamum indicum]
CL3121.Contig4	XP_012856870.1	86.13	1E-83	cytochrome P450 734A1 [Sesamum indicum]
CL15636.Contig1	XP_011093610.1	76.82	1.1E-213	cytochrome P450 716B1 [Sesamum indicum]
CL15636.Contig2	XP_011097890.1	76	1.1E-42	cytochrome P450 716B1 [Sesamum indicum]
CL1630.Contig10	XP_012853592.1	64.12	3.2E-143	cytochrome P450 [Theobroma cacao]
CL1630.Contig14	XP_012853592.1	63.06	3.9E-192	cytochrome P450 [Theobroma cacao]
CL1630.Contig15	XP_012853592.1	63.44	3.5E-193	cytochrome P450 [Theobroma cacao]
CL1630.Contig17	XP_012853592.1	63.32	5.8E-193	cytochrome P450 [Theobroma cacao]
CL1630.Contig6	XP_012853592.1	63.58	5.4E-194	cytochrome P450 [Theobroma cacao]
CL1630.Contig9	XP_012853592.1	63.06	1.5E-191	cytochrome P450 [Theobroma cacao]
CL16808.Contig2	XP_010252185.1	60.81	2.1E-170	cytochrome P450 [Macleaya cordata]
CL3302.Contig7	GAX81290.1	27.41	9.7E-38	cytochrome P450 711A1 [Ananas comosus]
CL3790.Contig1	XP_011070085.1	62.89	4.6E-183	cytochrome P450 94A1 [Sesamum indicum]

CL3790.Contig2	XP_011070085.1	63.48	1.4E-183	cytochrome P450 94A1 [Sesamum indicum]
Unigene7648	BAS30379.1	61.31	3.3E-127	cytochrome P450 [Ajuga reptans]
Unigene38195	AAL99201.1	87.38	3.2E-159	cytochrome P450 98A2 [Sesamum indicum]
CL7066.Contig3	AGN04218.1	64	5E-183	cytochrome P450 [Salvia miltiorrhiza]
CL7066.Contig4	AGN04218.1	64.81	4.3E-187	cytochrome P450 [Salvia miltiorrhiza]
CL7066.Contig7	AGN04218.1	64.85	4.5E-192	cytochrome P450 [Salvia miltiorrhiza]
CL7066.Contig8	AGN04218.1	64.62	5.8E-187	cytochrome P450 [Salvia miltiorrhiza]
CL7066.Contig9	AGN04218.1	67.09	4.2E-193	cytochrome P450 [Salvia miltiorrhiza]
CL4153.Contig10	AFZ40057.1	86.22	2E-203	cytochrome P450 [Ocimum basilicum]
CL4153.Contig6	AEX07772.1	81.55	1.3E-239	cytochrome P450 [Catharanthus roseus]
CL4153.Contig7	XP_012851292.1	86.31	3.3E-252	cytochrome P450 [Ocimum basilicum]
CL4346.Contig10	XP_012852863.1	55.37	1.4E-168	cytochrome P450 [Theobroma cacao]
CL4346.Contig4	XP_012852863.1	56.9	6.6E-173	cytochrome P450 [Theobroma cacao]
CL4346.Contig7	XP_012852863.1	55.93	2.1E-169	cytochrome P450 [Theobroma cacao]
CL4346.Contig8	XP_012852863.1	56.31	8.3E-171	cytochrome P450 [Theobroma cacao]
CL4861.Contig1	AOW42546.1	58.31	3.8E-90	cytochrome P450 76AK8 [Salvia rosmarinus]
CL4861.Contig2	AOW42546.1	62.2	2.5E-180	cytochrome P450 76AK8 [Salvia rosmarinus]
CL5138.Contig1	CDY56377.1	38.79	1.8E-12	CYP71B25 [Arabidopsis thaliana]
CL5583.Contig2	XP_011101836.1	75.11	3.5E-207	cytochrome P450 724B1 [Sesamum indicum]
CL5609.Contig2	CAA70575.1	72.3	5.9E-210	cytochrome P450 [Nepeta racemosa]
CL5609.Contig4	CAA70575.1	70.08	2.8E-204	cytochrome P450 [Nepeta racemosa]
CL5609.Contig5	CAA70575.1	70.39	5.7E-205	cytochrome P450 [Nepeta racemosa]
CL8195.Contig1	XP_011098098.1	81.84	3.1E-192	cytochrome P450 724B1 [Sesamum indicum]
CL8195.Contig3	XP_011098098.1	77.2	1.2E-216	cytochrome P450 724B1 [Sesamum indicum]
CL8195.Contig4	XP_011098098.1	77.04	4.7E-213	cytochrome P450 724B1 [Sesamum indicum]
CL8310.Contig1	EYU22015.1	43.77	6.9E-114	cytochrome P450 [Salvia miltiorrhiza]
CL8310.Contig2	AOW42546.1	66.11	3.3E-196	cytochrome P450 76AK8 [Salvia rosmarinus]
CL8745.Contig1	XP_011082379.1	84.48	5E-263	cytochrome P450 86A1 [Sesamum

				indicum]
CL8745.Contig3	XP_011082379.1	84.07	8.3E-261	cytochrome P450 86A1 [Sesamum indicum]
CL9036.Contig1	AGN04219.1	81.97	3E-18	cytochrome P450 [Salvia miltiorrhiza]
Unigene10634	CAD20576.2	87.4	5.6E-264	cytochrome P450 98A2 [Sesamum indicum]
Unigene13163	KZV52647.1	73.79	5.7E-208	CYP74A51 [Camellia sinensis]
Unigene26262	XP_011094380.1	73.45	7.7E-217	cytochrome P450 94A1 [Theobroma cacao]
Unigene3030	XP_011081026.1	58.75	9.7E-43	cytochrome P450 [Corchorus olitorius]
Unigene30346	XP_012852140.1	84.74	2.2E-90	cytochrome P450 704C1 [Sesamum indicum]
Unigene31465	NP_001291339.1	74.36	3E-231	cytochrome P450 98A3 [Sesamum indicum]
Unigene5513	ALM25789.1	91.67	1.6E-89	cytochrome P450 716A96 [Salvia pomifera]
CL10076.Contig1	KZV21240.1	64.59	3.8E-74	cytochrome P450 CYP89A115 [Salvia miltiorrhiza]
CL10107.Contig1	XP_011092252.1	80.2	3.6E-241	cytochrome P450 710A11 [Sesamum indicum]
CL10107.Contig3	XP_011085253.1	81.3	2.4E-243	cytochrome P450 710A11 [Sesamum indicum]
CL10107.Contig4	XP_011092252.1	80.73	7E-243	cytochrome P450 710A11 [Sesamum indicum]
CL10163.Contig1	AMZ03397.1	65.86	9.6E-175	cytochrome P450 CYP71AT85 [Plectranthus barbatus]
CL10163.Contig3	AJD25156.1	65.58	1.2E-174	cytochrome P450 CYP71AT92 [Salvia miltiorrhiza]
CL10601.Contig1	AJD25196.1	77.83	8.8E-184	cytochrome P450 CYP81Q40 [Salvia miltiorrhiza]
CL10601.Contig2	AJD25196.1	79.74	8.5E-211	cytochrome P450 CYP81Q40 [Salvia miltiorrhiza]
CL10743.Contig1	AJD25170.1	72.29	6.7E-223	cytochrome P450 CYP72A329 [Salvia miltiorrhiza]
CL10743.Contig2	NP_001291324.1	67.64	1.3E-207	cytochrome P450 CYP72A328 [Salvia miltiorrhiza]
CL10743.Contig3	AJD25170.1	71.12	1.4E-217	cytochrome P450 CYP72A329 [Salvia miltiorrhiza]

CL10743.Contig4	AJD25170.1	74.22	2E-228	cytochrome P450 CYP72A329 [Salvia miltiorrhiza]
CL10743.Contig5	NP_001291324.1	75.92	6.2E-155	cytochrome P450 CYP72A328 [Salvia miltiorrhiza]
CL10743.Contig6	AJD25170.1	74.42	2.2E-229	cytochrome P450 CYP72A329 [Salvia miltiorrhiza]
CL10743.Contig7	AJD25170.1	72.29	7.2E-223	cytochrome P450 CYP72A329 [Salvia miltiorrhiza]
CL10997.Contig1	XP_011095827.1	64.52	1.1E-196	cytochrome P450 CYP92B28 [Salvia miltiorrhiza]
CL11238.Contig1	AJD25202.1	72.93	2.7E-219	cytochrome P450 CYP82U4 [Salvia miltiorrhiza]
CL11238.Contig3	AJD25202.1	76.04	1.4E-138	cytochrome P450 CYP82U4 [Salvia miltiorrhiza]
CL11238.Contig4	AJD25202.1	72.85	1E-218	cytochrome P450 CYP82U4 [Salvia miltiorrhiza]
CL11719.Contig2	XP_012838752.1	60.72	2.2E-176	cytochrome P450 CYP76AH10 [Plectranthus barbatus]
CL11888.Contig1	ALM25795.1	74.56	4.4E-64	cytochrome P450 CYP76A35 [Salvia miltiorrhiza]
CL11888.Contig2	ALM25795.1	77.43	2.4E-97	cytochrome P450 CYP76A35 [Salvia miltiorrhiza]
CL11888.Contig3	AJD25179.1	78.17	7.5E-229	cytochrome P450 CYP76A35 [Salvia miltiorrhiza]
CL12095.Contig1	AJD25203.1	79.21	3.5E-243	cytochrome P450 CYP82V2 [Salvia miltiorrhiza]
CL1211.Contig1	AMZ03398.1	66.45	1.8E-114	cytochrome P450 CYP71AT87 [Plectranthus barbatus]
CL1211.Contig2	AMZ03398.1	71.84	8.8E-114	cytochrome P450 CYP71AT87 [Plectranthus barbatus]
CL1211.Contig3	AMZ03398.1	70.92	9.5E-114	cytochrome P450 CYP71AT87 [Plectranthus barbatus]
CL1211.Contig4	AMZ03398.1	70.16	2.1E-197	cytochrome P450 CYP71AT87 [Plectranthus barbatus]
CL1211.Contig5	AMZ03398.1	67.56	4.8E-200	cytochrome P450 CYP71AT87 [Plectranthus barbatus]
CL1211.Contig6	AMZ03398.1	69.96	2.2E-196	cytochrome P450 CYP71AT87 [Plectranthus barbatus]

CL12291.Contig2	AJD25184.1	80.44	7E-122	cytochrome P450 CYP76G16 [Salvia miltiorrhiza]
CL12553.Contig1	AJD25211.1	94.53	1.1E-257	cytochrome P450 CYP90A39 [Salvia miltiorrhiza]
CL12553.Contig2	AJD25211.1	94.29	1.3E-255	cytochrome P450 CYP90A39 [Salvia miltiorrhiza]
CL12553.Contig3	AJD25211.1	91.61	2.5E-165	cytochrome P450 CYP90A39 [Salvia miltiorrhiza]
CL12758.Contig1	AJD25180.1	75	2.8E-221	cytochrome P450 CYP76A36 [Salvia miltiorrhiza]
CL12758.Contig2	AJD25180.1	74.95	1.9E-219	cytochrome P450 CYP76A36 [Salvia miltiorrhiza]
CL1296.Contig2	AJD25208.1	91.78	4.7E-284	cytochrome P450 CYP86A92 [Salvia miltiorrhiza]
CL13202.Contig1	AJD25221.1	84.76	9.5E-243	cytochrome P450 CYP94C54 [Salvia miltiorrhiza]
CL13202.Contig2	AJD25221.1	84.76	7.6E-243	cytochrome P450 CYP94C54 [Salvia miltiorrhiza]
CL13512.Contig3	AJD25222.1	78.32	7E-215	cytochrome P450 CYP94C55 [Salvia miltiorrhiza]
CL13619.Contig1	AJD25147.1	92.28	4E-267	cytochrome P450 CYP51G1 [Salvia miltiorrhiza]
CL13619.Contig2	AJD25147.1	92.48	9.5E-268	cytochrome P450 CYP51G1 [Salvia miltiorrhiza]
CL13619.Contig3	AJD25147.1	92.48	1E-267	cytochrome P450 CYP51G1 [Salvia miltiorrhiza]
CL13790.Contig1	XP_012845193.1	75.8	6.4E-221	cytochrome P450 CYP736A122 [Salvia miltiorrhiza]
CL1384.Contig1	AJD25192.1	75.77	1.7E-80	cytochrome P450 CYP79D40 [Salvia miltiorrhiza]
CL1384.Contig3	AJD25192.1	73.63	2.8E-81	cytochrome P450 CYP79D40 [Salvia miltiorrhiza]
CL13862.Contig1	AJD25214.1	86.68	7.8E-181	cytochrome P450 CYP92A73 [Salvia miltiorrhiza]
CL13862.Contig2	AJD25214.1	92.19	6.4E-274	cytochrome P450 CYP92A73 [Salvia miltiorrhiza]
CL13862.Contig3	AJD25214.1	93.36	5.5E-112	cytochrome P450 CYP92A73 [Salvia miltiorrhiza]

CL13862.Contig4	AJD25214.1	92.23	6.3E-200	cytochrome P450 CYP92A73 [Salvia miltiorrhiza]
CL13862.Contig5	AJD25214.1	92.02	1.4E-111	cytochrome P450 CYP92A73 [Salvia miltiorrhiza]
CL14346.Contig1	AJD25155.1	72.34	2.5E-206	cytochrome P450 CYP71AT91 [Salvia miltiorrhiza]
CL14346.Contig2	AJD25155.1	71.17	2.7E-202	cytochrome P450 CYP71AT91 [Salvia miltiorrhiza]
CL14346.Contig3	AJD25155.1	68.21	9.6E-120	cytochrome P450 CYP71AT91 [Salvia miltiorrhiza]
CL14346.Contig4	AJD25155.1	75.68	1.4E-174	cytochrome P450 CYP71AT91 [Salvia miltiorrhiza]
CL14759.Contig1	AJD25220.1	83.76	4.2E-239	cytochrome P450 CYP94B50 [Salvia miltiorrhiza]
CL14759.Contig2	AJD25220.1	83.76	3.1E-239	cytochrome P450 CYP94B50 [Salvia miltiorrhiza]
CL14945.Contig1	AJD25227.1	89.48	3.6E-300	cytochrome P450 CYP97B34 [Salvia miltiorrhiza]
CL14945.Contig2	AJD25227.1	82.04	5.5E-283	cytochrome P450 CYP97B34 [Salvia miltiorrhiza]
CL15079.Contig1	AJD25247.1	79.42	2.1E-243	cytochrome P450 CYP714G14 [Salvia miltiorrhiza]
CL15079.Contig2	AJD25247.1	83.11	2.8E-254	cytochrome P450 CYP714G14 [Salvia miltiorrhiza]
CL15079.Contig4	AJD25247.1	74.91	6.8E-123	cytochrome P450 CYP714G14 [Salvia miltiorrhiza]
CL15149.Contig1	AHJ89438.1	80.33	8.8E-135	cytochrome P450 CYP93B25 [Salvia miltiorrhiza]
CL15149.Contig2	BAB59004.1	83.53	1.6E-246	cytochrome P450 CYP93B25 [Salvia miltiorrhiza]
CL15149.Contig3	BAB59004.1	84.33	4.2E-249	cytochrome P450 CYP93B25 [Salvia miltiorrhiza]
CL15249.Contig1	ALM25790.1	76.57	3.9E-213	cytochrome P450 CYP728D17 [Salvia miltiorrhiza]
CL15249.Contig2	ALM25790.1	77.04	1.2E-214	cytochrome P450 CYP728D17 [Salvia miltiorrhiza]
CL15547.Contig3	BAB59005.1	81.45	8.7E-249	cytochrome P450 CYP75B80 [Salvia miltiorrhiza]

CL16151.Contig1	AJD25177.1	86.45	1.3E-246	cytochrome P450 CYP75B79 [Salvia miltiorrhiza]
CL16151.Contig2	AJD25177.1	83.59	3.5E-250	cytochrome P450 CYP75B79 [Salvia miltiorrhiza]
CL16414.Contig1	AJD25231.1	87.13	1.9E-261	cytochrome P450 CYP98A77 [Salvia miltiorrhiza]
CL16524.Contig1	AJD25156.1	81.07	1.3E-110	cytochrome P450 CYP71AT92 [Salvia miltiorrhiza]
CL16698.Contig1	XP_012844328.1	78.01	6E-61	cytochrome P450 CYP72A330 [Salvia miltiorrhiza]
CL16698.Contig2	AJD25171.1	79.43	5.9E-63	cytochrome P450 CYP72A330 [Salvia miltiorrhiza]
CL16700.Contig1	AJD25237.1	85.14	5.7E-260	cytochrome P450 CYP706C35 [Salvia miltiorrhiza]
CL16700.Contig2	AJD25237.1	84.94	2E-259	cytochrome P450 CYP706C35 [Salvia miltiorrhiza]
CL16700.Contig3	AJD25237.1	84.94	3.6E-146	cytochrome P450 CYP706C35 [Salvia miltiorrhiza]
CL16744.Contig1	AJD25175.1	86.67	1.4E-62	cytochrome P450 CYP74B21 [Salvia miltiorrhiza]
CL16744.Contig2	AJD25175.1	84.68	3.6E-238	cytochrome P450 CYP74B21 [Salvia miltiorrhiza]
CL1708.Contig1	AJD25225.1	72.29	3E-218	cytochrome P450 CYP96A85 [Salvia miltiorrhiza]
CL1708.Contig2	AJD25225.1	71.49	2E-214	cytochrome P450 CYP96A85 [Salvia miltiorrhiza]
CL1708.Contig3	AJD25225.1	71.54	5.7E-214	cytochrome P450 CYP96A85 [Salvia miltiorrhiza]
CL17240.Contig2	XP_011100951.1	66.25	4.5E-154	cytochrome P450 CYP84A60 [Salvia miltiorrhiza]
CL17371.Contig1	AJD25171.1	81.43	1.1E-250	cytochrome P450 CYP72A330 [Salvia miltiorrhiza]
CL17371.Contig2	AJD25171.1	74.13	2.8E-226	cytochrome P450 CYP72A330 [Salvia miltiorrhiza]
CL17371.Contig3	AJD25171.1	81.1	4.2E-55	cytochrome P450 CYP72A330 [Salvia miltiorrhiza]
CL17371.Contig5	AJD25171.1	81.43	1.1E-250	cytochrome P450 CYP72A330 [Salvia miltiorrhiza]

CL17371.Contig7	AJD25171.1	80.8	5.7E-97	cytochrome P450 CYP72A330 [Salvia miltiorrhiza]
CL17371.Contig8	AJD25171.1	82.63	2.7E-195	cytochrome P450 CYP72A330 [Salvia miltiorrhiza]
CL17371.Contig9	AJD25171.1	82.77	9.7E-170	cytochrome P450 CYP72A330 [Salvia miltiorrhiza]
CL17586.Contig2	ABC75596.1	94.03	1.5E-107	cytochrome P450 CYP73A120 [Salvia miltiorrhiza]
CL17745.Contig1	XP_011082968.1	67.95	9.7E-212	cytochrome P450 CYP72A326 [Salvia miltiorrhiza]
CL17745.Contig10	XP_011082968.1	73.79	9.8E-183	cytochrome P450 CYP72A330 [Salvia miltiorrhiza]
CL17745.Contig11	XP_011082968.1	69.05	4.7E-217	cytochrome P450 CYP72A326 [Salvia miltiorrhiza]
CL17745.Contig2	XP_011082968.1	68.02	6.8E-210	cytochrome P450 CYP72A330 [Salvia miltiorrhiza]
CL17745.Contig3	XP_011082968.1	73.69	1.7E-227	cytochrome P450 CYP72A330 [Salvia miltiorrhiza]
CL17745.Contig4	XP_011082968.1	69.77	2.2E-219	cytochrome P450 CYP72A330 [Salvia miltiorrhiza]
CL17745.Contig6	XP_011082968.1	70.74	2.5E-220	cytochrome P450 CYP72A330 [Salvia miltiorrhiza]
CL17745.Contig7	XP_011082968.1	70.6	4.9E-218	cytochrome P450 CYP72A330 [Salvia miltiorrhiza]
CL17745.Contig8	XP_011082968.1	70.78	9.4E-214	cytochrome P450 CYP72A330 [Salvia miltiorrhiza]
CL17745.Contig9	AJD25171.1	74.85	2.2E-232	cytochrome P450 CYP72A330 [Salvia miltiorrhiza]
CL17775.Contig1	AMZ03386.1	71.17	3.1E-201	cytochrome P450 CYP71D381 [Plectranthus barbatus]
CL1797.Contig2	XP_011095827.1	71.37	8.3E-207	cytochrome P450 CYP92B29 [Salvia miltiorrhiza]
CL2184.Contig1	AJD25205.1	83.17	9.3E-252	cytochrome P450 CYP84A61 [Salvia miltiorrhiza]
CL2184.Contig2	AJD25205.1	83.82	5.5E-252	cytochrome P450 CYP84A61 [Salvia miltiorrhiza]
CL2184.Contig3	AJD25205.1	83.24	3.1E-250	cytochrome P450 CYP84A61 [Salvia miltiorrhiza]

CL2184.Contig4	AJD25204.1	88.22	4.9E-264	cytochrome P450 CYP84A60 [Salvia miltiorrhiza]
CL2184.Contig5	AJD25204.1	88.42	1.1E-264	cytochrome P450 CYP84A60 [Salvia miltiorrhiza]
CL2184.Contig6	AJD25205.1	83.01	5.5E-141	cytochrome P450 CYP84A61 [Salvia miltiorrhiza]
CL2184.Contig7	AJD25204.1	83.95	2.1E-249	cytochrome P450 CYP84A60 [Salvia miltiorrhiza]
CL2184.Contig8	AJD25205.1	83.2	7.1E-252	cytochrome P450 CYP84A61 [Salvia miltiorrhiza]
CL2340.Contig1	AJD25245.1	78.39	6.9E-243	cytochrome P450 CYP714E21 [Salvia miltiorrhiza]
CL2795.Contig1	AJD25249.1	84.97	4.1E-247	cytochrome P450 CYP716C12 [Salvia miltiorrhiza]
CL2795.Contig2	AJD25249.1	86.34	2.6E-248	cytochrome P450 CYP716C12 [Salvia miltiorrhiza]
CL2795.Contig3	AJD25249.1	91.84	7E-51	cytochrome P450 CYP716C12 [Salvia miltiorrhiza]
CL2957.Contig4	AMZ03392.1	79.18	9.1E-227	cytochrome P450 CYP76AH8 [Plectranthus barbatus]
CL2957.Contig8	AMZ03392.1	67.01	1.4E-187	cytochrome P450 CYP76AH8 [Plectranthus barbatus]
CL3067.Contig1	AMZ03386.1	64.75	1.7E-98	cytochrome P450 CYP71D381 [Plectranthus barbatus]
CL3249.Contig1	AJD25261.1	74.36	1.8E-222	cytochrome P450 CYP749A39 [Salvia miltiorrhiza]
CL3249.Contig2	AJD25261.1	70.33	4.3E-93	cytochrome P450 CYP749A39 [Salvia miltiorrhiza]
CL3249.Contig3	AJD25261.1	70.74	7.1E-154	cytochrome P450 CYP749A39 [Salvia miltiorrhiza]
CL3249.Contig4	AJD25261.1	74.63	4.2E-114	cytochrome P450 CYP749A39 [Salvia miltiorrhiza]
CL3249.Contig5	AJD25261.1	72.8	5.4E-217	cytochrome P450 CYP749A39 [Salvia miltiorrhiza]
CL3249.Contig6	AJD25261.1	69.67	8E-209	cytochrome P450 CYP749A39 [Salvia miltiorrhiza]
CL3359.Contig1	AJD25199.1	77.02	3.7E-222	cytochrome P450 CYP81Q43 [Salvia miltiorrhiza]

CL3359.Contig5	AJD25199.1	77.42	6.9E-222	cytochrome P450 CYP81Q43 [Salvia miltiorrhiza]
CL3585.Contig2	AJD25169.1	68.79	1.4E-58	cytochrome P450 CYP72A328 [Salvia miltiorrhiza]
CL3766.Contig2	XP_011100884.1	72.87	6.5E-211	cytochrome P450 CYP736A123 [Salvia miltiorrhiza]
CL3868.Contig10	AJD25242.1	89.16	1E-166	cytochrome P450 CYP707A102 [Salvia miltiorrhiza]
CL3868.Contig11	AJD25242.1	80.65	2E-192	cytochrome P450 CYP707A102 [Salvia miltiorrhiza]
CL3868.Contig12	AJD25242.1	80.56	3E-192	cytochrome P450 CYP707A102 [Salvia miltiorrhiza]
CL3868.Contig13	AJD25242.1	87.82	1.3E-235	cytochrome P450 CYP707A102 [Salvia miltiorrhiza]
CL3868.Contig14	AJD25242.1	86.53	2E-215	cytochrome P450 CYP707A102 [Salvia miltiorrhiza]
CL3868.Contig15	AJD25242.1	84.85	6.1E-168	cytochrome P450 CYP707A102 [Salvia miltiorrhiza]
CL3868.Contig17	AJD25242.1	90.85	9.1E-84	cytochrome P450 CYP707A102 [Salvia miltiorrhiza]
CL3868.Contig2	AJD25242.1	87.77	2E-235	cytochrome P450 CYP707A102 [Salvia miltiorrhiza]
CL3868.Contig3	AJD25242.1	87.21	8.7E-217	cytochrome P450 CYP707A102 [Salvia miltiorrhiza]
CL3868.Contig4	AJD25242.1	86.65	9.9E-193	cytochrome P450 CYP707A102 [Salvia miltiorrhiza]
CL3868.Contig5	AJD25242.1	87.16	1.7E-216	cytochrome P450 CYP707A102 [Salvia miltiorrhiza]
CL3868.Contig6	AJD25242.1	87.16	1.7E-216	cytochrome P450 CYP707A102 [Salvia miltiorrhiza]
CL3868.Contig9	AJD25242.1	91.02	2.9E-211	cytochrome P450 CYP707A102 [Salvia miltiorrhiza]
CL4153.Contig1	AJD25248.1	88.73	7.2E-262	cytochrome P450 CYP716A89 [Salvia miltiorrhiza]
CL4153.Contig2	AJD25248.1	91.04	3E-266	cytochrome P450 CYP716A89 [Salvia miltiorrhiza]
CL4153.Contig3	AJD25248.1	87.82	1.7E-203	cytochrome P450 CYP716A89 [Salvia miltiorrhiza]

CL4153.Contig4	AMZ03376.1	94	1.7E-52	cytochrome P450 CYP716A68 [Plectranthus barbatus]
CL4153.Contig5	AMZ03376.1	90	3.8E-50	cytochrome P450 CYP716A68 [Plectranthus barbatus]
CL4683.Contig1	AJD25174.1	80.17	7E-164	cytochrome P450 CYP74A1 [Salvia miltiorrhiza]
CL4683.Contig2	AJD25174.1	81.73	8.5E-252	cytochrome P450 CYP74A1 [Salvia miltiorrhiza]
CL4683.Contig3	AJD25174.1	81.92	1.5E-251	cytochrome P450 CYP74A1 [Salvia miltiorrhiza]
CL4774.Contig1	AJD25156.1	64.69	9.2E-193	cytochrome P450 CYP71AT92 [Salvia miltiorrhiza]
CL4774.Contig2	AMZ03397.1	67.8	1.3E-196	cytochrome P450 CYP71AT85 [Plectranthus barbatus]
CL4774.Contig3	AOA52647.1	67.13	1.5E-195	cytochrome P450 CYP71AT92 [Salvia miltiorrhiza]
CL4774.Contig4	AMZ03397.1	67.59	6.3E-187	cytochrome P450 CYP71AT85 [Plectranthus barbatus]
CL542.Contig1	AJD25168.1	67.06	1.1E-85	cytochrome P450 CYP72A327 [Salvia miltiorrhiza]
CL542.Contig10	AJD25168.1	65.06	3.9E-83	cytochrome P450 CYP72A327 [Salvia miltiorrhiza]
CL542.Contig2	AJD25168.1	70.82	6E-134	cytochrome P450 CYP72A327 [Salvia miltiorrhiza]
CL542.Contig3	AJD25168.1	72.58	2.3E-102	cytochrome P450 CYP72A327 [Salvia miltiorrhiza]
CL542.Contig4	AJD25168.1	76.36	1.2E-111	cytochrome P450 CYP72A327 [Salvia miltiorrhiza]
CL542.Contig5	AJD25168.1	75.98	2E-228	cytochrome P450 CYP72A327 [Salvia miltiorrhiza]
CL542.Contig6	AJD25168.1	73.6	4.6E-228	cytochrome P450 CYP72A327 [Salvia miltiorrhiza]
CL542.Contig7	AMZ03389.1	80.14	8.8E-61	cytochrome P450 CYP72A294 [Plectranthus barbatus]
CL542.Contig9	AJD25168.1	77.13	2.6E-79	cytochrome P450 CYP72A327 [Salvia miltiorrhiza]
CL548.Contig2	AJD25163.1	63.56	7.3E-182	cytochrome P450 CYP71D410 [Salvia miltiorrhiza]

CL548.Contig3	AJD25163.1	58.36	3E-85	cytochrome P450 CYP71D410 [Salvia miltiorrhiza]
CL548.Contig4	AMZ03386.1	53.6	5.8E-27	cytochrome P450 CYP71D381 [Plectranthus barbatus]
CL548.Contig5	AJD25163.1	62.65	3.6E-177	cytochrome P450 CYP71D410 [Salvia miltiorrhiza]
CL5600.Contig1	XP_011078642.1	78.71	4.3E-231	cytochrome P450 CYP736A121 [Salvia miltiorrhiza]
CL5600.Contig2	AJD25256.1	78.99	5.7E-233	cytochrome P450 CYP736A121 [Salvia miltiorrhiza]
CL5600.Contig3	AJD25256.1	75.4	8.7E-224	cytochrome P450 CYP736A121 [Salvia miltiorrhiza]
CL5609.Contig3	O04164.1	69.13	2.2E-202	cytochrome P450 CYP71A57 [Salvia miltiorrhiza]
CL6335.Contig1	AJD25156.1	75.05	1.3E-218	cytochrome P450 CYP71AT92 [Salvia miltiorrhiza]
CL6481.Contig2	AMZ03395.1	73.28	1.1E-222	cytochrome P450 CYP82D63 [Plectranthus barbatus]
CL6672.Contig1	XP_012853764.1	79.47	8.5E-84	cytochrome P450 CYP81Q43 [Salvia miltiorrhiza]
CL6909.Contig1	XP_019163354.1	70.67	1.7E-227	cytochrome P450 [Cynara cardunculus var. scolymus]
CL6909.Contig2	XP_020546889.1	72.5	2.4E-229	cytochrome P450 [Cynara cardunculus var. scolymus]
CL699.Contig3	AJD25218.1	72.08	8.5E-210	cytochrome P450 CYP94A48 [Salvia miltiorrhiza]
CL699.Contig6	AJD25218.1	83.26	5.9E-231	cytochrome P450 CYP94A48 [Salvia miltiorrhiza]
CL699.Contig7	AJD25218.1	82.63	2.1E-228	cytochrome P450 CYP94A48 [Salvia miltiorrhiza]
CL699.Contig8	AJD25218.1	80.31	6.8E-236	cytochrome P450 CYP94A48 [Salvia miltiorrhiza]
CL699.Contig9	AJD25218.1	79.92	1E-234	cytochrome P450 CYP94A48 [Salvia miltiorrhiza]
CL7255.Contig1	EYU32284.1	84.51	2E-138	cytochrome P450 CYP78A115 [Salvia miltiorrhiza]
CL7255.Contig2	EYU32284.1	73.08	4.2E-219	cytochrome P450 CYP78A115 [Salvia miltiorrhiza]

CL7255.Contig3	EYU32284.1	73.27	2.7E-219	cytochrome P450 CYP78A115 [Salvia miltiorrhiza]
CL7373.Contig2	AJD25228.1	89.34	1.1E-279	cytochrome P450 CYP97C28 [Salvia miltiorrhiza]
CL7416.Contig1	AJD25229.1	90.06	2E-187	cytochrome P450 CYP98A75 [Salvia miltiorrhiza]
CL7416.Contig2	AJD25229.1	92.29	2.5E-280	cytochrome P450 CYP98A75 [Salvia miltiorrhiza]
CL7416.Contig3	AJD25229.1	87.4	4E-188	cytochrome P450 CYP98A75 [Salvia miltiorrhiza]
CL7416.Contig6	AJD25229.1	91.93	5.6E-188	cytochrome P450 CYP98A75 [Salvia miltiorrhiza]
CL7416.Contig7	AJD25229.1	92.14	9.8E-282	cytochrome P450 CYP98A75 [Salvia miltiorrhiza]
CL7416.Contig8	AJD25229.1	91.55	7.1E-280	cytochrome P450 CYP98A75 [Salvia miltiorrhiza]
CL7517.Contig1	AJD25193.1	76.24	2.1E-225	cytochrome P450 CYP81B61 [Salvia miltiorrhiza]
CL7517.Contig2	AJD25193.1	76.13	3.3E-225	cytochrome P450 CYP81B61 [Salvia miltiorrhiza]
CL7517.Contig3	AJD25193.1	76.57	2.5E-128	cytochrome P450 CYP81B61 [Salvia miltiorrhiza]
CL7517.Contig4	AJD25193.1	75.74	3.1E-127	cytochrome P450 CYP81B61 [Salvia miltiorrhiza]
CL7706.Contig2	XP_013723179.1	26.76	2E-09	cytochrome P450 CYP83A1 [Brassica oleracea var. italica]
CL843.Contig1	AJD25210.1	82.31	8.2E-242	cytochrome P450 CYP89A115 [Salvia miltiorrhiza]
CL843.Contig2	AJD25210.1	83.23	1.8E-244	cytochrome P450 CYP89A115 [Salvia miltiorrhiza]
CL843.Contig4	AJD25210.1	81.7	1.4E-174	cytochrome P450 CYP89A115 [Salvia miltiorrhiza]
CL843.Contig5	AJD25210.1	82.3	4.2E-234	cytochrome P450 CYP89A115 [Salvia miltiorrhiza]
CL8878.Contig1	AJD25187.1	90.62	1.8E-271	cytochrome P450 CYP77A27 [Salvia miltiorrhiza]
CL9408.Contig1	AJD25198.1	80.96	8.8E-236	cytochrome P450 CYP81Q42 [Salvia miltiorrhiza]

CL9408.Contig2	AJD25198.1	80.96	9E-236	cytochrome P450 CYP81Q42 [Salvia miltiorrhiza]
CL9914.Contig2	AJD25185.1	76.16	8.3E-215	cytochrome P450 CYP76S7 [Salvia miltiorrhiza]
CL9914.Contig5	AJD25185.1	75.35	3.4E-216	cytochrome P450 CYP76S7 [Salvia miltiorrhiza]
CL9950.Contig2	ABC75596.1	95.45	6.7E-284	cytochrome P450 CYP73A120 [Salvia miltiorrhiza]
Unigene10759	AJD25169.1	77.34	6.2E-190	cytochrome P450 CYP72A328 [Salvia miltiorrhiza]
Unigene11470	AJD25169.1	64.67	2.1E-129	cytochrome P450 CYP72A328 [Salvia miltiorrhiza]
Unigene11591	XP_011082223.1	80.6	8.5E-250	cytochrome P450 711A1 isoform X2 [Sesamum indicum]
Unigene11871	AJD25221.1	82.35	9.6E-58	cytochrome P450 CYP94C54 [Salvia miltiorrhiza]
Unigene12093	AJD25226.1	93.65	0	cytochrome P450 CYP97A41 [Salvia miltiorrhiza]
Unigene12109	AJD25235.1	82.02	6.4E-241	cytochrome P450 CYP704A99 [Salvia miltiorrhiza]
Unigene12411	AJD25189.1	78.04	3.4E-147	cytochrome P450 CYP78A113 [Salvia miltiorrhiza]
Unigene13158	AMZ03392.1	76.02	2.5E-107	cytochrome P450 CYP76AH8 [Plectranthus barbatus]
Unigene23370	AJD25215.1	73.36	4.3E-216	cytochrome P450 CYP92B28 [Salvia miltiorrhiza]
Unigene2359	AMZ03391.1	86.17	3.8E-252	cytochrome P450 CYP76AH9 [Plectranthus barbatus]
Unigene23619	XP_011082968.1	73.01	1.4E-225	cytochrome P450 CYP72A330 [Salvia miltiorrhiza]
Unigene24431	XP_011070908.1	75	3.7E-226	cytochrome P450 CYP749A22 [Sesamum indicum]
Unigene28242	XP_011089169.1	78.54	3.6E-110	cytochrome P450 CYP716D20 [Plectranthus barbatus]
Unigene2848	AMZ03402.1	67.17	1.1E-125	cytochrome P450 CYP76AH15 [Plectranthus barbatus]
Unigene299	AJD25159.1	79.04	2.6E-232	cytochrome P450 CYP71AU52 [Salvia miltiorrhiza]

Unigene29940	XP_011085047.1	72.97	6.5E-37	cytochrome P450 CYP71AU51 [Salvia miltiorrhiza]
Unigene301	ALR74628.1	84.39	3.7E-259	cytochrome P450 CYP701A40 [Salvia miltiorrhiza]
Unigene31829	ALM25793.1	67.89	1.3E-94	cytochrome P450 CYP71AU53 [Salvia miltiorrhiza]
Unigene3203	AJD25219.1	79.17	6.5E-229	cytochrome P450 CYP94A49 [Salvia miltiorrhiza]
Unigene3291	AJD25190.1	79.4	1E-235	cytochrome P450 CYP78A114 [Salvia miltiorrhiza]
Unigene34382	XP_012850388.1	56.52	1.1E-61	cytochrome P450 CYP716A89 [Salvia miltiorrhiza]
Unigene34383	AHA50080.1	61.75	2.4E-76	cytochrome P450 [Eleutherococcus senticosus]
Unigene36145	AJD25156.1	77.14	3.6E-40	cytochrome P450 CYP71AT92 [Salvia miltiorrhiza]
Unigene36533	XP_001690122.1	24.23	2.5E-16	cytochrome P450 [Chlamydomonas reinhardtii]
Unigene38087	AJD25199.1	75.37	1.4E-86	cytochrome P450 CYP81Q43 [Salvia miltiorrhiza]
Unigene52691	AJD25218.1	83.02	1.1E-45	cytochrome P450 CYP94A48 [Salvia miltiorrhiza]
Unigene52945	ALR74628.1	76.18	2.3E-155	cytochrome P450 CYP701A40 [Salvia miltiorrhiza]
Unigene54571	XP_011087220.1	71.76	1.6E-213	cytochrome P450 CYP94A48 [Salvia miltiorrhiza]
Unigene56325	XP_011100232.1	75.37	1.7E-145	cytochrome P450 CYP76A36 [Salvia miltiorrhiza]
Unigene57565	AJD25168.1	82.16	8.3E-189	cytochrome P450 CYP72A327 [Salvia miltiorrhiza]
Unigene57566	AJD25168.1	81.89	1.2E-108	cytochrome P450 CYP72A327 [Salvia miltiorrhiza]
Unigene57706	XP_011082968.1	75.93	2.7E-117	cytochrome P450 CYP72A294 [Plectranthus barbatus]
Unigene58003	AMZ03392.1	86.7	2E-116	cytochrome P450 CYP76AH8 [Plectranthus barbatus]
Unigene5881	XP_011101640.1	77.2	6.9E-236	cytochrome P450 CYP77A27 [Salvia miltiorrhiza]

Unigene61608	AJD25192.1	72.39	1.2E-219	cytochrome P450 CYP79D40 [Salvia miltiorrhiza]
Unigene6317	AJD25240.1	83.26	2.2E-212	cytochrome P450 CYP707A100 [Salvia miltiorrhiza]
Unigene6321	AJD25167.1	72.83	5E-221	cytochrome P450 CYP72A326 [Salvia miltiorrhiza]
Unigene6536	AJD25197.1	78.09	1E-220	cytochrome P450 CYP81Q41 [Salvia miltiorrhiza]
Unigene6689	XP_011099350.1	70.25	7.4E-197	cytochrome P450 CYP81Q43 [Salvia miltiorrhiza]
Unigene7148	AJD25175.1	83.71	4E-165	cytochrome P450 CYP74B21 [Salvia miltiorrhiza]
Unigene7220	Q42716.1	82.34	5.5E-230	cytochrome P450 CYP71A58 [Salvia miltiorrhiza]
Unigene8586	AJD25260.1	84.78	1.3E-255	cytochrome P450 CYP749A38 [Salvia miltiorrhiza]
Unigene8669	AJD25207.1	91.56	8.6E-297	cytochrome P450 CYP86A91 [Salvia miltiorrhiza]
Unigene89	AJD25234.1	81.64	4.5E-249	cytochrome P450 CYP704A98 [Salvia miltiorrhiza]
Unigene90	AJD25234.1	81.64	3.1E-249	cytochrome P450 CYP704A98 [Salvia miltiorrhiza]
Unigene9229	AJD25251.1	83.98	3.3E-238	cytochrome P450 CYP720A1 [Salvia miltiorrhiza]
Unigene9597	AJD25169.1	68.28	2.1E-84	cytochrome P450 CYP72A328 [Salvia miltiorrhiza]

Supplementary Table S3 Glucosyltransferases annotated in *C. chinense*.

Query_id	Subject_id	Identity	E_value	Subject_annotation
CL13036.Contig2	XP_012858542.1	72.38	8.9E-209	glycosyltransferase [Lycium barbarum]
CL13535.Contig1	XP_011099399.1	80.56	7.6E-127	glycosyltransferase 6 [Sesamum indicum]
CL8216.Contig4	XP_020535976.1	56.45	1.2E-06	glycosyl transferase [Macleaya cordata]
Unigene12918	BAG31948.1	76.38	3.7E-201	UGT88D7 [Perilla frutescens]
Unigene12917	BAG31948.1	75.82	1.3E-201	UGT88D7 [Perilla frutescens]
Unigene31451	XP_012858542.1	77.51	2.6E-72	glycosyltransferase [Lycium barbarum]
Unigene801	XP_012845190.1	67.92	3E-184	UDP-rhamnose:rhamnosyltransferase 1 [Dorcoceras hygrometricum]
Unigene7805	XP_012839050.1	54.39	2.3E-134	UDP-glycosyltransferase 74E2 [Morus notabilis]
Unigene6530	XP_012843823.1	67.8	2E-190	UDP-sugar:glycosyltransferase [Forsythia x intermedia]
Unigene58585	XP_011098502.1	58.75	3.9E-75	UDP-glycosyltransferase 90A1 [Sesamum indicum]
Unigene54111	XP_011088227.1	59.07	1.5E-157	UDP glucosyltransferase [Scutellaria barbata]
Unigene53347	XP_011087901.1	79.94	3E-155	UDP-glucuronosyl/UDP-glucosyltransferase [Macleaya cordata]
Unigene33854	XP_011084237.1	71.69	1E-110	UDP-glycosyltransferase 91C1 [Sesamum indicum]
Unigene32667	XP_011077327.1	77.01	4.6E-73	UDP-glycosyltransferase 86A1 [Sesamum indicum]
Unigene3258	XP_012831918.1	74.7	9.5E-137	Glycosyl transferase [Dorcoceras hygrometricum]
Unigene319	XP_011082325.1	63.58	3.9E-180	UDP-glycosyltransferase 85A28 [Vitis vinifera]
Unigene3061	XP_011098740.1	63.47	2.3E-168	UDP-glycosyltransferase 83A1 [Sesamum indicum]
Unigene30402	XP_011098517.1	83.41	0	xyloglucan glycosyltransferase 4 [Sesamum indicum]
Unigene28067	XP_011100452.1	60.04	2.1E-156	UDP-glycosyltransferase 71E1 [Sesamum indicum]
Unigene24073	XP_012894366.1	30.43	1.5E-18	glycosyl transferase [Blastocystis sp. subtype 4]
Unigene22910	XP_011076757.1	64.62	6.4E-176	udp-glycosyltransferase 85a7 [Nicotiana attenuata]

Unigene22140	XP_011101335.1	87.01	4.1E-228	UDP-glucuronosyl/UDP-glucosyltransferase [Corchorus olitorius]
Unigene19903	XP_011098502.1	60.55	4.4E-90	UDP-glycosyltransferase 90A1 [Sesamum indicum]
Unigene11429	KZV42770.1	57.05	1.7E-92	UDP-glycosyltransferase 83A1 [Morus notabilis]
Unigene11150	XP_011089226.1	66.36	1.1E-36	UDP-glycosyltransferase 87A1-like [Sesamum indicum]
Unigene10594	Q9ZR27.1	77.07	6.2E-204	UDP-glucose:glucosyltransferase [Lycium barbarum]
Unigene10175	XP_011100441.1	71.62	1.3E-183	UDP-glycosyltransferase 82A1 [Sesamum indicum]
CL9711.Contig1	XP_019055273.1	49.88	1.2E-108	UDP-glycosyltransferase 23 [Pueraria montana var. lobata]
CL9658.Contig1	XP_011094738.1	66.94	6.5E-93	UDP-glycosyltransferase 86A1-like [Sesamum indicum]
CL8978.Contig2	XP_011094571.1	67.69	2.5E-193	UDP-glycosyltransferase 73C5 [Sesamum indicum]
CL8978.Contig1	XP_011094571.1	66.05	1.7E-190	UDP-glycosyltransferase 73C5 [Sesamum indicum]
CL8850.Contig3	XP_020548990.1	73.2	4.1E-106	UDP-glycosyltransferase 88B1-like [Sesamum indicum]
CL8850.Contig2	XP_020548989.1	72.14	3.7E-117	UDP-glycosyltransferase 88B1-like [Sesamum indicum]
CL7672.Contig5	XP_011088204.1	70.93	1.5E-203	UDP-glycosyltransferase 84A22 [Camellia sinensis]
CL7672.Contig4	XP_011088204.1	70.93	1.9E-203	UDP-glycosyltransferase 84A22 [Camellia sinensis]
CL7672.Contig3	XP_011088204.1	72.73	1.2E-206	UDP-glycosyltransferase 84A22 [Camellia sinensis]
CL7672.Contig1	XP_011088204.1	71.91	5.1E-204	UDP-glycosyltransferase 84A22 [Camellia sinensis]
CL753.Contig2	XP_011075343.2	54.73	3.6E-57	UDP-glucuronosyl/UDP-glucosyltransferase [Cynara cardunculus var. scolymus]
CL7267.Contig4	XP_011082054.1	71.09	4.5E-191	UDP-glycosyltransferase 74E2-like [Sesamum indicum]
CL7267.Contig3	XP_011082054.1	67.84	3.1E-187	UDP-glycosyltransferase 74E2-like [Sesamum indicum]

CL7267.Contig1	XP_011082054.1	71.02	2.8E-188	UDP-glycosyltransferase 74E2-like [Sesamum indicum]
CL6566.Contig2	XP_011093112.2	54.94	4.7E-144	UDP-glycosyltransferase 91C1 [Sesamum indicum]
CL6566.Contig1	XP_011093112.2	56.09	7.8E-145	UDP-glycosyltransferase 91C1 [Sesamum indicum]
CL5761.Contig4	XP_011094737.1	66.74	6.7E-183	UDP-glycosyltransferase 86A1-like [Sesamum indicum]
CL5446.Contig2	XP_011094568.1	76.34	2.3E-214	UDP-glycosyltransferase 73D1 [Sesamum indicum]
CL5446.Contig1	XP_011094568.1	75.51	6.4E-214	UDP-glycosyltransferase 73D1 [Sesamum indicum]
CL5412.Contig3	XP_011076757.1	69.42	3.2E-196	UDP-glycosyltransferase 85a7 [Nicotiana attenuata]
CL5412.Contig2	XP_011076757.1	72.52	6.1E-207	UDP -glycosyltransferase 85a7 [Nicotiana attenuata]
CL4477.Contig1	XP_011084237.1	71.46	5.1E-198	UDP-glycosyltransferase 91C1 [Sesamum indicum]
CL3150.Contig4	XP_012858058.1	63.22	5.7E-144	UDP-glycosyltransferase BMGT2 [Bacopa monnieri]
CL3150.Contig2	XP_012858058.1	59.71	1.2E-110	UDP-glycosyltransferase BMGT2 [Bacopa monnieri]
CL3150.Contig1	XP_012858058.1	66.18	1.3E-150	UDP-glycosyltransferase BMGT2 [Bacopa monnieri]
CL2908.Contig5	XP_012855183.1	59.15	6E-42	UDP -glycosyltransferase 708c1 [Nicotiana attenuata]
CL1905.Contig6	XP_011092852.1	68.09	6.1E-186	UDP-glycosyltransferase 91C1 [Sesamum indicum]
CL1905.Contig5	XP_011092852.1	68.3	8.1E-188	UDP-glycosyltransferase 91C1 [Sesamum indicum]
CL1905.Contig4	XP_011092852.1	72.9	3.5E-88	UDP-glycosyltransferase 91C1 [Sesamum indicum]
CL1905.Contig3	XP_011092852.1	69.68	2.2E-196	UDP-glycosyltransferase 91C1 [Sesamum indicum]
CL1905.Contig2	XP_011092852.1	69.39	3.6E-140	UDP-glycosyltransferase 91C1 [Sesamum indicum]
CL1905.Contig1	XP_011092852.1	67.2	3E-92	UDP-glycosyltransferase 91C1 [Sesamum indicum]

CL17415.Contig1	XP_011089086.1	77.97	2.6E-209	UDP-glycosyltransferase 87A1-like [Sesamum indicum]
CL16513.Contig2	XP_011098517.1	87.35	0	xyloglucan glycosyltransferase 4 [Sesamum indicum]
CL16513.Contig1	XP_011085811.1	86.51	0	xyloglucan glycosyltransferase 4 [Sesamum indicum]
CL16251.Contig2	XP_011084980.1	70.87	6.7E-195	UDP-glycosyltransferase 76F1 [Jatropha curcas]
CL15953.Contig2	XP_011077373.2	66.37	9.5E-121	glycosyl transferase [Dorcoceras hygrometricum]
CL15953.Contig1	XP_011077373.2	66.67	3.7E-120	glycosyl transferase [Dorcoceras hygrometricum]
CL15287.Contig2	XP_012829432.1	68.13	2.2E-145	UDP-glucuronosyl/UDP-glucosyltransferase [Macleaya cordata]
CL13535.Contig3	XP_011099399.1	77.49	7.6E-222	UDP-glucuronosyl/UDP-glucosyltransferase [Macleaya cordata]
CL13502.Contig1	AJM89728.1	71.24	7E-189	UDP-glucosyltransferase [Leonurus japonicus]
CL12824.Contig1	XP_012836028.1	70.45	4.6E-199	UDP-glycosyltransferase 86A1 [Sesamum indicum]
CL11215.Contig3	XP_011086492.1	74.91	2.8E-239	UDP-glycosyltransferase 74B1 [Sesamum indicum]
CL11215.Contig2	XP_011086492.1	74.74	1.1E-238	UDP-glycosyltransferase 74B1 [Sesamum indicum]
CL10742.Contig1	BAI65913.1	53.51	3E-134	UDP-sugar:glycosyltransferase [Forsythia x intermedia]
CL10593.Contig9	XP_011073993.1	58.3	1.7E-144	UDP-glycosyltransferase 74E2-like [Sesamum indicum]
CL10593.Contig7	XP_012839050.1	55.63	9.1E-133	UDP-glycosyltransferase BMGT1 [Bacopa monnieri]
CL10593.Contig6	XP_011073993.1	61.71	2.5E-153	UDP-glycosyltransferase 74E2-like [Sesamum indicum]
CL10593.Contig5	XP_011073993.1	55.18	3E-133	UDP-glycosyltransferase 74E2-like [Sesamum indicum]
CL10593.Contig4	XP_011073993.1	54.55	1.3E-108	UDP-glycosyltransferase 74E2-like [Sesamum indicum]
CL10593.Contig3	XP_011073993.1	61.27	3.3E-152	UDP-glycosyltransferase 74E2-like [Sesamum indicum]

CL10593.Contig2	XP_011073993.1	59.24	5.4E-144	UDP-glycosyltransferase 74E2-like [Sesamum indicum]
CL10593.Contig11	XP_012839050.1	54.75	3.9E-131	UDP-glycosyltransferase BMGT1 [Bacopa monnieri]
CL10593.Contig10	XP_011073993.1	63.89	3.1E-70	UDP-glycosyltransferase 74E2-like [Sesamum indicum]
CL10046.Contig2	XP_011070892.1	67.86	3.4E-186	UDP-glycosyltransferase 89A2 [Sesamum indicum]
CL10046.Contig1	XP_011070892.1	65.62	5.4E-153	UDP-glycosyltransferase 89A2 [Sesamum indicum]
CL10006.Contig4	XP_011078914.1	48.1	2.2E-08	UDP-glucuronosyl/UDP-glucosyltransferase [Corchorus capsularis]

Supplementary Table S4 Beta amyrin synthase annotated in *C. chinense*.

Query id	Subject id	Identity	E value	Subject annotation
CL17709.Contig1	XP_011093795.1	83.38	0.00E+00	beta-amyrin synthase [Sesamum indicum]
CL2196.Contig2	KZV14753.1	87.05	0.00E+00	beta-amyrin synthase [Dorcoceras hygrometricum]
CL2196.Contig4	KZV14753.1	86.26	0.00E+00	beta-amyrin synthase [Dorcoceras hygrometricum]
CL2196.Contig5	KZV14753.1	86.26	0.00E+00	beta-amyrin synthase [Dorcoceras hygrometricum]
CL2196.Contig8	KZV14753.1	86.39	0.00E+00	beta-amyrin synthase [Dorcoceras hygrometricum]
CL5601.Contig1	KZV51042.1	83.01	0.00E+00	beta-amyrin synthase [Dorcoceras hygrometricum]

Supplementary Table S5 GO terms from biological process ontology for leaf versus root in *C. chinense*.

Gene Ontology term	Genome frequency of use (25024)	Corrected P-value
photosynthesis	265 (1.1%)	2.00E-12
cell wall organization or biogenesis	688 (2.7%)	1.74E-10
carbohydrate metabolic process	2029 (8.1%)	3.61E-10
cellular polysaccharide metabolic process	390 (1.6%)	8.81E-10
polysaccharide metabolic process	631 (2.5%)	1.13E-09
cell wall macromolecule metabolic process	184 (0.7%)	3.28E-09
external encapsulating structure organization	582 (2.3%)	8.17E-09
cell wall organization	575 (2.3%)	9.96E-09
cellular glucan metabolic process	341 (1.4%)	2.33E-08

Supplementary Table S6 GO terms from biological process ontology for stem versus root in *C. chinense*.

Gene Ontology term	Genome frequency of use (25024)	Corrected P-value
drug catabolic process	332 (1.3%)	5.54e-20
hydrogen peroxide metabolic process	153 (0.6%)	6.64e-15
photosynthesis	265 (1.1%)	8.96e-15
hydrogen peroxide catabolic process	152 (0.6%)	1.38e-14
cofactor catabolic process	201 (0.8%)	7.78e-14
reactive oxygen species metabolic process	176 (0.7%)	1.13e-13
cell wall organization or biogenesis	688 (2.7%)	4.36e-11
cellular polysaccharide metabolic process	390 (1.6%)	2.63e-10
polysaccharide metabolic process	631 (2.5%)	8.29e-10

Supplementary Table S7 GO terms from molecular function ontology for leaf versus root in *C. chinense*.

Gene Ontology term	Genome frequency of use (40108)	Corrected P-value
oxidoreductase activity	4432 (11.1%)	1.03e-17
structural molecule activity	1459 (3.6%)	1.50e-11
tetrapyrrole binding	959 (2.4%)	4.26e-09
structural constituent of ribosome	1200 (3.0%)	6.53e-09
copper ion binding	260 (0.6%)	2.35e-08
xyloglucan:xyloglucosyl transferase activity	89 (0.2%)	3.28e-08
cofactor binding	2538 (6.3%)	7.60e-07
peroxidase activity	279 (0.7%)	8.42e-07
heme binding	890 (2.2%)	9.59e-07

Supplementary Table S8 GO terms from molecular function ontology for stem versus root in *C. chinense*.

Gene Ontology term	Genome frequency of use (40108)	Corrected P-value
oxidoreductase activity	4432 (11.1%)	2.74e-35
tetrapyrrole binding	959 (2.4%)	5.17e-16
DNA binding transcription factor activity	1235 (3.1%)	1.11e-13
heme binding	890 (2.2%)	9.69e-12
cofactor binding	2538 (6.3%)	3.56e-09
transcription regulator activity	1427 (3.6%)	4.92e-09
oxidoreductase activity, acting on peroxide as acceptor	295 (0.7%)	1.44e-07
hydrolase activity, hydrolyzing O-glycosyl compounds	964 (2.4%)	5.69e-07
peroxidase activity	279 (0.7%)	9.09e-07

Supplementary Table S9 RNA information of different tissues.

Sample	Concentration (ng/μL)	Total amount (μg)	OD260/280	RIN	28S/18S
Leaves	395	11.85	2.03	7.5	1.6
Roots	497	14.91	2.00	8.0	1.5
Stems	226	6.78	1.99	9.5	1.7

Supplementary Table S10 Genes and primer sequences used for RT-qPCR.

Gene ID	Primers (bp)	Length (bp)	Tm (℃)
CL2016.Contig3	5′-TTGACCATGGAGCACAGAGT-3′	20	56.2
	5′-TAGAACAACAGTCGCTGCAA-3′	20	55.1
CL10703. Contig3	5′-TGGACCACAGACACTCGACA-3′	20	55.9
	5′-ACCTGATCAGCTAGGAGGGA-3′	20	57.3
CL10042.Contig3	5'-AACTTCAGAAAGGAGTGGCA-3'	20	54.7
	5'-ATCTCGGTGAAGCACACCTC-3'	20	57.1
CL8457.Contig10	5'-ATAAAGTCATCATTGGGGGC-3'	20	56.5
	5'-TTGGTAGTGGTTTTGACGTG-3'	20	54.1
CL10625.Contig5	5'-TTCTCCGCTTAAATCTCTCC-3'	20	54.1
	5'-ATGGAGTTGGATTTGGATTT-3'	20	53.6
CL7015.Contig4	5-GTACGAAGTCAACTGCGTTA-3'	20	51.3
	5'-GGTGATGCATTGTTCCCTCT-3'	20	57.0
β-actin	5′-ACGAGCTTCCTGATGGACAA-3′	20	56.2
	5′-TTCCAGCAGCTTCCATACCA-3′	20	56.3
